# Effects of dietary exposure to plant toxins on bioaccumulation, survival, and growth of black soldier fly (*Hermetia illucens*) larvae and lesser mealworm (*Alphitobius diaperinus*)

**DOI:** 10.1016/j.heliyon.2024.e26523

**Published:** 2024-02-16

**Authors:** Patrick P.J. Mulder, Judith T.L. Mueller-Maatsch, Nathan Meijer, Marlou Bosch, Lisa Zoet, H.J. Van Der Fels-Klerx

**Affiliations:** a Wageningen Food Safety Research (WFSR), Part of Wageningen University and Research, Akkermaalsbos 2, 6708 WB Wageningen, the Netherlands; b Ynsect NL (formerly Protifarm), Harderwijkerweg 141a, 3852 AB Ermelo, the Netherlands; c Bestico, Industrieweg 6, 2651 BE Berkel en Rodenrijs, the Netherlands

**Keywords:** Black soldier fly larvae (*Hermetia illucens*), Lesser mealworm (*Alphitobius diaperinus*), Pyrrolizidine alkaloids, Tropane alkaloids, Transfer, LC-MS/MS

## Abstract

In their natural habitat, insects may bioaccumulate toxins from plants for defence against predators. When insects are accidently raised on feed that is contaminated with toxins from co-harvested herbs, this may pose a health risk when used for human or animal consumption. Plant toxins of particular relevance are the pyrrolizidine alkaloids (PAs), which are genotoxic carcinogens produced by a wide variety of plant species and the tropane alkaloids (TAs) which are produced by a number of Solanaceae species. This study aimed to investigate the transfer of these plant toxins from substrates to black soldier fly larvae (BSFL) and lesser mealworm (LMW). PAs and the TAs atropine and scopolamine were added to insect substrate simulating the presence of different PA- or TA-containing herbs, and BSFL and LMW were grown on these substrates. Bioaccumulation from substrate to insects varied widely among the different plant toxins. Highest bioaccumulation was observed for the PAs europine, rinderine and echinatine. For most PAs and for atropine and scopolamine, bioaccumulation was very low. In the substrate, PA N-oxides were quickly converted to the corresponding tertiary amines. More research is needed to verify the findings of this study at larger scale, and to determine the potential role of the insect and/or substrate microbiome in metabolizing these toxins.

## Highlights

• Black soldier fly larvae and lesser mealworm were exposed to dietary plant toxins.
• No significant effects on survival/yield for either species.
• Little transfer for most tested toxins, but accumulation for some.
• Apparent degradation of toxins by insects, but possibly also by substrate microbiome.

## 1 Introduction

Insects are increasingly being used as food and feed ingredients in Europe. Two types of insects that are of particular interest are black soldier fly larvae (BSFL, *Hermetia illucens* L., 1758 (Diptera: Stratiomyidae)) and lesser mealworm (LMW, *Alphitobius diaperinus* Panzer, 1797 (Coleoptera: Tenebrionidae)) [1,2]. Both species have been approved by the European Commission for use in animal feed [[3], [4], [5]] (Commission Regulation (EU) 2017/893) and LMW have recently also been concluded to be safe as food for human consumption by EFSA [6] and approved for marketing as a novel food (Commission Implementing Regulation (EU) 2023/58). Therefore, as long as good manufacturing procedures such as hazard analysis critical control points (HACCP) are followed, and the substrate is of feed-grade quality, these insects produced for food or feed can generally be considered be safe for consumption.

With the scaling up to insect mass production, the quality of the substrate becomes more and more important [7]. In addition to nutritional levels for optimal growth and quality of the insect larvae, the safety of the substrate is crucial. Of particular relevance is the possible presence of microbiological and chemical (contaminants) hazards that could transfer from the substrate into the insect larvae, and possibly bioaccumulate [8].

In recent years, the amount of research on the safety of insects for food and feed has increased tremendously. Contaminants that have been investigated include heavy metals [9,10], mycotoxins [11,12], environmental contaminants [13], and pesticides [14,15]. Nevertheless, one important group of contaminants has not been investigated yet: plant toxins, even though certain plant toxins are known to accumulate in insects feeding on them [[16], [17], [18]]. The presence of plant toxins in substrate used for insects for food and feed may firstly result into the insect accumulating these substances and/or the insects converting the compounds from the substrate into toxic metabolites [[19], [20], [21]]. Some plant toxins – in particular 1,2-unsaturated pyrrolizidine alkaloids (Fig. S1) – are genotoxic, and the consumption of insects with even low doses of these compounds should therefore be avoided [[22], [23], [24], [25]]. Secondly, the growth and/or survival of the reared insects could be affected by the presence of plant toxins in substrates. Such adverse effects have, for instance, been observed for the presence of certain insecticide residues in the substrate – for both BSFL and LMW [14,15].

Commercial feeds for BSFL and LMW differ between the two species, but in general the diets consist of a variety of feed materials of vegetable origin [26,27]. Herbs that produce plant toxins may be present in or near the fields from which the feed ingredients are harvested and may unintentionally end up in the feed products. Herbs that are frequently implicated in food contamination incidents are for instance: common ragwort (*Jacobaea vulgaris* Gaertn. Asteraceae), common groundsel (*Senecio vulgaris* L. Asteraceae), narrow-leaved ragwort (*Senecio inaequidens* DC. Asteraceae), viper's bugloss (*Echium vulgare* L. Boraginaceae), common heliotrope (*Heliotropium europaeum* L. Boraginaceae) [19], and thorn apple/Jimson weed (*Datura stramonium* L. Solanaceae) [28,29].

In addition to more traditional feed materials, insect farmers are looking into certain waste streams that may be unsuitable as feed for conventional livestock species (e.g., poultry, pigs) but could be utilized by these insect species. However, especially in the case of such novel feed ingredients for insects, contaminants that would be of less concern for conventional feed sources may be present and be relevant to insects. For instance, potato skins (member of the genus *solanum*) may contain high levels of steroidal alkaloids, such as α-solanine and α-chaconine [30], which necessitates extensive processing before they are suitable for use as feed for conventional livestock [31].

In Europe, legal limits (maximum limits, MLs) for the presence of several groups of plant toxins in food and feed have been established (Commission Regulation (EU) 2023/935; Commission Directive 2002/32/EC). Specific MLs have been set for the maximum concentration of the toxin in a particular feed/food product (toxin/product combinations).

The objective of this study was to investigate if the presence of plant toxins in the substrate (feed) affects the growth and survival of BSFL and LMW, and if the toxins are bioaccumulated or metabolically converted by these insect species. We hypothesized that either, or both, species would be affected in terms of survival and/or growth; and that some of the plant toxins would bioaccumulate in the larval biomass. As far as we are aware, this is the first study to experimentally assess the effects of plant toxins on insects reared for food and feed purposes.

## 2 Materials and methods

### 2.1 Materials

The feed for BSFL from Meelfabriek De Jongh (Steenbergen, the Netherlands) contained primarily wheat bran, while for LMW the feed consisted of wheat middlings with vitamins, minerals and a plant-based protein concentrate mixed by Research Diet Services (RDS, Wijk bij Duurstede, The Netherlands). A single batch of BSFL and LMW feed material was used throughout the study, to control for any batch-specific differences. None of the plant toxins investigated in this study were detected in the feed materials.

A total of 59 pyrrolizidine alkaloids (PAs) and tropane alkaloids (TAs) analytical standards were used in this study. The standards comprised the 35 PAs and 2 TAs included in the current EU legislation (Regulation (EU) 2023/935), 14 additional standards that were relevant for this study [19] and 10 standards that were used as internal standard (IS). Details on vendors, purity and CAS numbers can be found in Table S1. The purity of the standards was at least 95%. Individual PA stock solutions of 200 μg/mL were prepared in methanol. For LC-MS/MS analyses, mixed PA solutions in methanol (5 μg/mL, 500 ng/mL) were prepared from the individual stocks. One set of solutions contained the PA free bases (tertiary amines, PAFBs) and the two TAs, one set contained the PA N-oxides (PANOs) and one set the internal standards. Methanol and acetonitrile, both of LC-MS grade, were obtained from Actu-All (Oss, The Netherlands). Both formic acid and ammonium carbonate were of analytical grade and purchased from Sigma-Aldrich (Zwijndrecht, The Netherlands). Deionized water (Milli-Q, Merck Millipore) with a minimum resistance of 18.2 M was used as solvent.

### 2.2 Feed preparation

Feed materials with specific toxin compositions were prepared by mixing blank BSFL and LMW insect feed with a spiking solution of plant toxins in methanol. Blank feed was ground with a GM 200 mill (Grindomix, Retsch, Haan, Germany) at 10.000 rpm for 1 min to achieve a particle size of approximately 1 mm. The feed was spiked to simulate the concentrations of PAs that were reported by Mulder et al. (2016) in feed for laying hens containing 0.5% or 0.1% of different herbs (Table 1) [19]. The concentrations of TAs simulated the inclusion of 0.1% Datura seeds. The feed for each treatment and each insect species was prepared in separate batches. Using commercially available PA standards, between 90% and 98% of the total composition of these herbs could be simulated. In treatment 3 (T3) (*S. inaequidens*) senkirkine was added in excess, to match the other PAs containing an otonecine base, for which no standards were available. See Table S2 for the composition of the spiked feed. To achieve homogenous distribution of the spiking solutions in each batch, a slurry consisting of 1 kg of feed, 67 ml of methanolic spike solution and 1–1.5 l methanol was mixed using a table-top mixer (Topcraft, the Netherlands). The mixture was then transferred to aluminum plates that were placed in a fume hood to allow the methanol to evaporate overnight. The resulting feed cake was loosened up using a kitchen spoon and ground (Grindomix, Retsch, Haan, Germany) at 10.000 rpm for 1 min.

**Table 1:** Prepared feeds, spiked with mixed PA standards to simulate PA-containing herbs, tropane alkaloids and controls.

Treatment No.	Treatment (species name)	Common name	Aimed total plant toxin concentration
T1	Simulated *Jacobaea vulgaris* mix	Common ragwort	5.4 mg/kg
T2	Simulated *Senecio vulgaris* mix	Common groundsel	10.5 mg/kg
T3	Simulated *Senecio inaequidens* mix	Narrow-leaved ragwort	54.6 mg/kg
T4	Simulated *Echium vulgare* mix	Viper's bugloss	5.5 mg/kg
T5	Simulated *Heliotropium europaeum* mix	Common heliotrope	19.7 mg/kg
T6	Simulated 5 herbs mix		19.1 mg/kg
T7	Atropine		4.0 mg/kg
T8	Scopolamine		1.0 mg/kg
T9	Blank feed, treated with methanol		–

### 2.3 Feeding trial black soldier fly larvae (BSFL)

The BSFL were reared on the different feed substrates (T1-T9) (Table 1) in two consecutive phases (P) with three replicates per treatment. The BSFL were provided by the research & development department of commercial BSFL-rearing organization Bestico B.V. (Berkel en Rodenrijs, The Netherlands), where the BSFL experiment took place. For both phases, the respective feed was mixed with lukewarm tap water (35% wheat bran to 65% water w/w) right before feeding and the containers used for both phases were distributed randomly over trays, which were stacked and then placed at 28 °C and 65% RH for 7 days. For P1 (day 0-day 8), 1000 g of wet feed was placed in a container (16,5 cm x 16,5 cm x 12,5 cm, Jokey, Wipperfürth, Germany) and 0.50 g of freshly hatched larvae (∼27.000 individuals of <24 h old) were spread on top. On day 5, a few ml of water was sprinkled on top of the diet to prevent crust forming.

For P2 (day 8-day 15), 50 g of wet feed was placed in a cylindrical petri dish with a fine mesh ventilation in the center of the lid (diameter 10 cm, height 4 cm, SPL Life Sciences, Gyeonggi-do, South Korea). A total of 100 individual larvae from each of the three replicates for all nine treatments (T1-T9) of P1 were moved to these replicate cylindrical petri dish. The 8-day-old larvae from the corresponding treatment and replicates were first weighed collectively and then placed into petri-dishes. After each phase, the larvae were separated from the residue by dry sieving and/or use of a tweezer.

The total number of live larvae harvested in P1 was calculated by dividing their total weight by individual average weight. The latter was determined by stirring the harvested larvae and then counting three subsamples of ∼1.0 g per replica. Live P2 larvae were rinsed, dried with a paper towel and then weighed and counted. All larvae were killed by freezing and stored at −18 °C until further analysis.

### 2.4 Feeding trial lesser mealworm (LMW)

LMW were reared for 21 days on spiked feed T1-T8 (Table 1) and methanol-treated control feed T9. Each treatment was performed in three replicates. Per replicate, 400 mg of first instar LMW (∼5900 individuals) were transferred to a separate container (PP, 187 × 120 × 80 mm, Dampack International, Werkendam, The Netherlands) and incubated at 30 °C and 55% RH. The LMW were provided by the research & development department of commercial LMW-rearing organization Ynsect NL (Ermelo, The Netherlands), where the LMW experiment took place. The respective feed was mixed with lukewarm tap water (50% feed to 50% water w/w) right before feeding. Feed was supplied daily during the experiment. From day 0 until day 7, around 10 g wet feed per day was supplied, increasing to 20 g wet feed on days 8–10, 30 g between day 11 and 13, 40 g on days 14–19 and 30 g on day 20. After 21 days, LMW were separated from the substrate by dry sieving as described in Meijer et al. [14], weighed and frozen, and a representative sample of each replicate was counted two or three times. The total number of post-experiment larvae per replicate was extrapolated from the count in the respective sample and the total yield. The LMW were killed by freezing and stored at −18 °C until further analysis.

### 2.5 Stability trial

The stability of the plant toxins under the incubation conditions was assessed in a feed stability trial in which the feed materials were mixed with water and incubation conditions of the insect trials were simulated. The conditions of the BSFL trial were simulated by transferring 2 × 2.0 g of substrate T1-T8 to 50-ml PP tubes. Water was added to the tubes in a ratio 35% feed to 65% water (w/w) (see 2.4). Two slightly different conditions were used: of one of the replicates, the screw cap of the tube was closed airtight, while for the other one the cap was placed but not closed. This was done to simulate different humidity conditions. The tubes were placed in an oven (T 6, Heraeus, Hanau, Germany) kept at 28 °C for 7 days. The conditions of the LMW trial were simulated by adding incremental portions of feed and water in a 1:1 ratio (see section 2.5) to the test tubes for a period of 3 weeks to a total of 2 × 2.5 g of substrate T1-T8. All feed samples were freeze dried, ground and homogenized before analysis (see section 2.7).

### 2.6 Processing of feed, larvae and residue samples

Samples (∼5–10 g) of feed, larvae and residual material were placed in 50 ml PP tubes and freeze dried (7 days, −55 °C) in a bench top freeze-dryer (Zirbus Vaco 5, Bad Grund, Germany). The dry matter (DM) content was determined by weighing the tubes before and after freeze-drying and the results can be found in Table S3. Feed samples were loosened up manually with a spoon, while the larvae were crushed to a fine powder using a pestle and mortar. Of the feed material of each treatment T1-T9 (Table 1), three aliquots of 2.0 ± 0.05 g were weighed into 50 ml PP tubes. To these tubes, 40 μl IS mix (5 μg/ml) was added, followed by 40 ml extraction solvent (0.2% formic acid solution). Samples were extracted for 30 min on a rotary tumbler (Heidolph Reax 2, Schwabach, Germany) and centrifuged for 15 min at 3000 g (Thermo Scientific SL 40R, Waltham, USA). From each supernatant an aliquot of 500 μl and of 50 μl were transferred to 500 μl mini UniPrep filtervials (Whatman, GE Healthcare, Maidstone, UK). The latter aliquot was diluted 10-fold by adding 450 μl of a 10% methanol in water solution.

Of each residue sample, 1.0 g was weighed into 50 ml PP tubes. 20 μl IS mix (5 μg/ml) was added, followed by 20 ml extraction solvent. Sample processing was performed as described for feed.

Of the larvae samples 0.5 g was weighed into 12 ml PP tubes. 10 μl IS mix (5 μg/ml) was added, followed by 10 ml extraction solvent. Sample processing was performed as described for feed. Finally, 1 ml aliquots were additionally filtered through 30 kD membrane filters (Amicon Ultra-4, Ultracel-30K, Merck Millipore, Carrigtwohill, Ireland).

### 2.7 LC-MS/MS analysis

Analysis was performed using an LC-MS/MS system consisting of a Waters Acquity UPLC coupled to a Xevo TQ-S tandem mass spectrometer (Waters, Milford, MA, USA). Two to three multiple reaction monitoring (MRM) transitions were measured per analyte in positive electrospray ionization (ESI) mode. The ion source parameters were set as follows: capillary voltage, 3.0 kV; source temperature, 150 °C; source offset, 60 V; desolvation temperature, 600 °C; cone gas flow, 150 l/h; desolvation gas flow, 800 l/h; collision-induced dissociation (CID) gas, argon, 4.3 × 10-3 mbar (0.17 ml/min). See Table S4 for detailed information on MRM transitions, MS settings and retention times.

The analytes were separated on an Acquity UPLC BEH C18 1.7 μm 150 × 2.1 mm column (Waters, Milford, MA, USA). The solvents used were (A) ammonium carbonate buffer (10 mmol/l, pH 9.0 ± 0.1) and (B) acetonitrile. At a flow rate of 0.4 ml/min, the linear gradient conditions were: 0.0 min, 0% B; 0.1 min, 5% B; 3.0 min, 10% B; 7.0 min, 24% B; 9.0 min, 30% B; 12.0 min, 70% B; 12.1 min–14.2 min, 0% B. The injection volume was 2 μl, the column oven temperature 50 °C. MassLynx 4.2 and TargetLynx XS software (Waters, Milford, MA, USA) were used for, respectively, data acquisition and processing.

### 2.8 Quantification and analytical quality control

Quantification was performed by means of matrix matched calibration curves prepared by fortifying blank control (T9) feed, residue or larvae. Samples were spiked before extraction with the mixed standard solutions of 500 ng/ml and 5 μg/l to produce 8-point calibration lines (0–1000 μg/kg for feed and residue and 0–500 μg/kg for larvae. Concentrations were corrected for IS response (See Table S4 for the specific IS used).

Calibration lines were run before and after the samples and the lines were combined for quantification. The linearity of the calibration curves (>0.990) was checked over two working ranges: a low range (first 6 points) and the full range (8 points). Concentrations falling within the low range were quantified with the 6-point calibration line, while concentrations exceeding the low calibration line were quantified with the full range line. When the concentration exceeded the full working range, the diluted extract was used for quantification of the specific compound.

Extraction efficiency (recovery) was monitored by spiking two blank sample extracts of feed, residue or larvae with 12.5 μl of a mixture of the PA and TA standards (500 ng/ml) corresponding to a level of 250 μg/kg. The Limit of Quantification (LoQ) was 5 μg/kg for feed and residue and 2.5 μg/kg for larvae.

### 2.9 Statistical analysis of survival and growth

Survival rate was calculated as the number of insects at the end of the respective farming phase (mean of 3 samples, as elaborated in sections 2, 2.4.5) in respect to the estimated number of larvae at the beginning. The yield was evaluated as the mean total biomass in g at the end of the rearing phase. Mean individual larval weight was expressed in mg and calculated as the total biomass of the sample divided by the number of counted larvae. Statistical analyses were conducted using SPSS Statistics for Microsoft Windows (version 25.0.0.2, IBM Corp., Armonk, NY, United States). The treatments were performed in triplicate so tests on conformity to a distribution type were not warranted [14,15]. Hence, non-parametric statistical tests were performed to determine statistical significance of findings. Significant effects on survival or growth were analyzed using a Kruskal-Wallis test with a significance level of 0.05. If differences were significant, a Mann-Whitney U was used as a post-hoc test (α = 0.05) in which the results of the control were compared against each treatment, with a significance level of 0.01 to account for multiple comparisons.

### 2.10 Calculation of mass balance and bioaccumulation factors

Plant toxin mass balance calculations were performed in accordance with Meijer et al. [15], as follows: the concentrations of toxins determined in, respectively, feed, larvae and residue (expressed as μg/kg DM) were multiplied by the weight of the samples (kg DM), yielding the total amount (μg) in each fraction. Recovery was expressed as percentage of the total amount of the toxins present in the larvae or residue sample compared to the starting feed material. To explore the uptake of toxins from the feed to the insects, bioaccumulation factors (BAFs) were calculated, by dividing the toxin concentration (expressed as μg/kg DM) in the larvae by that in the feed/residue that they were exposed to. This ratio provides an indication whether the toxin tends to concentrate in the larvae relative to the feed they are exposed to. Transfer of toxins was calculated by dividing the total amount of toxins present in the larvae by the amount in the part of the feed that was consumed. The total amount of plant toxins accumulated in the BSFL P1 was found to be negligible and is therefore not corrected for regarding the transfer of BSFL from P1 to P2.

## 3 Results

### 3.1 Homogeneity and stability of plant toxins in feed

A test feed material spiked with 10 Pas was prepared, showing that the toxins were homogenously distributed (Table S5). The relative standard deviation (RSD) ranged from 2.9% to 11.1% and the recovery between 85% and 110%.

The measured concentrations in the final feed materials are shown in Table S6. Generally, the concentrations determined in the feeds were in line with the theoretically spiked toxin amounts. However, overall, a slight increase in the total amount of PAFBs and a decrease in the PANOs was noted, particularly for the BSFL feeds; suggesting that the PANOs were not fully stable under the conditions of feed preparation. Furthermore, it was observed that in T1 and T6, jaconine and jaconine-NO were present in higher concentrations than intended – the latter had not even been added. Both could be artifacts formed from jacobine or jacobine-NO by reaction of chloride ion with the epoxide ring. For further calculations, the concentrations measured from the spiked feed were used.

**Fig. 1:**
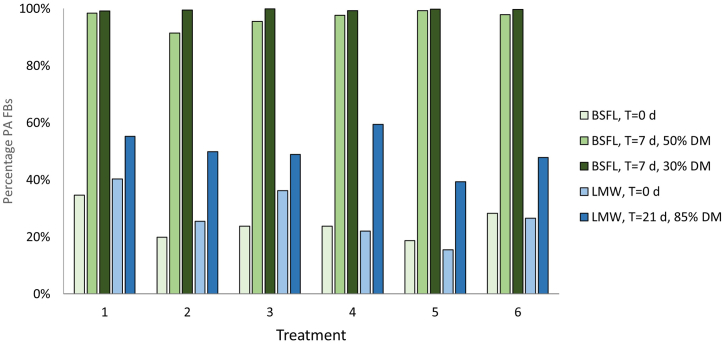
Effect of incubation conditions on the pyrrolizidine alkaloid (PA) composition in the absence of insects at different dry matter (DM) content values: the percentage of Pas free bases (PAFBs) present in the starting materials and after 7 days of incubation (BSFL feeds) or 21 days (LMW feeds), for treatments T1-T6. T1: Simulated J. vulgaris mix, T2: Simulated *S. vulgaris* mix, T3: Simulated S. inaequidens mix, T4: Simulated *E. vulgare* mix, T5: Simulated H. europaeum mix, T6: Simulated 5 herbs mix.

In terms of the stability of the analytical standards used for spiking the feed material; for the feeds containing atropine or scopolamine, the impact of the incubation was limited. However, the incubation had a strong impact on the composition of the Pas in the feed materials (Fig. 1; Table S7). In the BSFL feeds with the lids closed, after one week of incubation the PANOs had almost completely disappeared (less than 1% remaining), and in the BSFL feeds with the loose caps the levels of PANOs were reduced to less than 10%. In the LMW feeds, the contribution of the PANOs varied between 40 and 60% of the total, compared to 60–85% at the start of the incubation. A concomitant increase in concentration of the PAFBs was observed for almost all feeds and conditions. The PAFB concentration in the BSFL feeds increased by 62% (T1) to 526% (T5). For the LMW feed, the concentration of PAFBs decreased in T1 (12% loss) and T3 (5% loss) but increased by 44–165% in the other treatments. In BSFL T1, T2, T3 and T6 the total concentration of Pas decreased during the incubation by 30–45%, but this decline was not seen for T4 and T5. In LMW T1, T2, T3 and T6 the total PA concentration declined by 20–35%, and in T4 and T5 by 10% or less (Table S7). Because the PA composition of the feeds changed substantially during the incubations, the calculations on bioaccumulation and transfer were based on the composition of the feed residues at the end of the incubations.

### 3.2 Yield and survival of insects on feed spiked with plant toxins

**Fig. 2:**
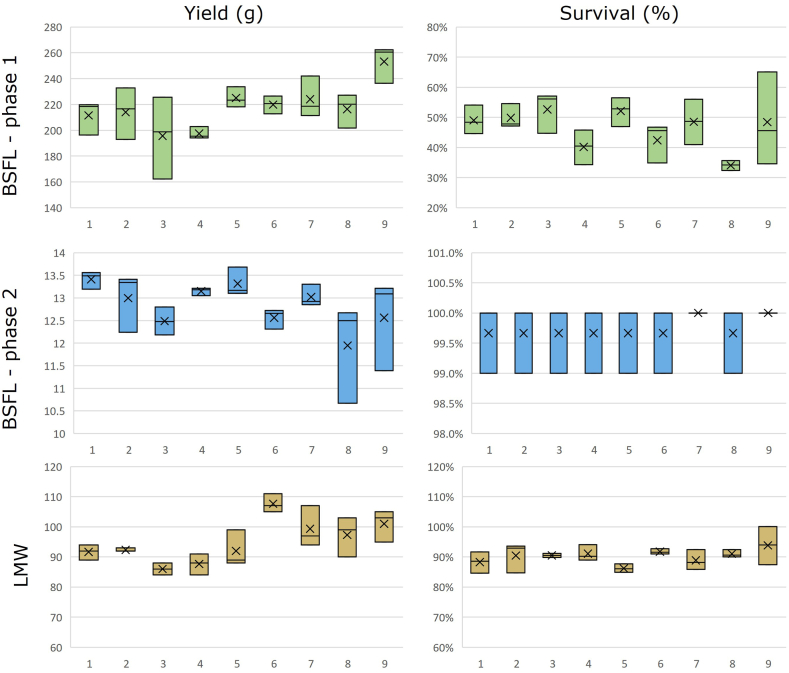
Box-plot showing yield (g FW) and survival (%) of lesser mealworm and black soldier fly larvae (Phase 1 and 2) for treatments T1-T9. T1: Simulated J. vulgaris mix, T2: Simulated *S. vulgaris* mix, T3: Simulated S. inaequidens mix, T4: Simulated *E. vulgare* mix, T5: Simulated H. europaeum mix, T6: Simulated 5 herbs mix, T7: Atropine, T8: Scopolamine, T9: Solvent control (methanol). Mean of N = 3 replicates.

The survival and yield of insects on the respective feeds gives insight in the effects of the respective plant toxins on insect production. Results in terms of survival and yield for LMW and BSFL (both phases) are shown in Fig. 2 and Table S8. For both BSFL experiments, no significant differences on survival and yield between treatments were seen (Kruskal-Wallis test, P > 0.05). For LMW differences between treatments were not significant for survival (Kruskal-Wallis test, P > 0.05), but they were significant for yield (P ≤ 0.05). However, the post-hoc test for yield did not find significant differences between any of the treatments and the control (Mann-Whitney *U* test, P > 0.01).

**Fig. 3:**
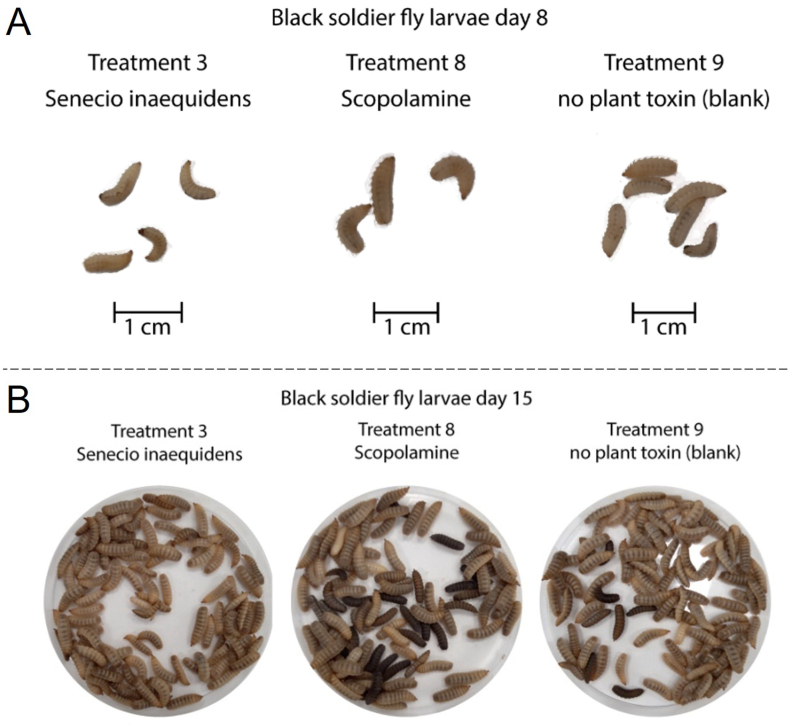
A: Black soldier fly larvae on day 8 (Phase 1) grown on treatments T3, T8 and T9, showing differences in growth; B: Black soldier fly larvae after day 15 (Phase 2) from the treatments T3, T8 and T9, showing differences in the number of prepupal larvae (dark coloured).

In the experiment with BSFL P1, it was observed that the larvae in T3 developed slower and were about 1–3 days behind the development of larvae of the other treatments. In T3 only 33% of the feed was consumed while in the other treatments a feed consumption of 47–53% was observed, these similar to the control (58%) (Table S9). However, the larvae to feed mass ratio was not lower for T3 (0.197) as compared to the other treatments (0.196–0.215) and the control (0.235). The low feed consumption in T3 was in line with the delayed growth of the larvae compared to the other treatments. Because the larvae of the P1 experiment would also be used in the P2 experiment, this slow development led us to prolong the previously planned farming period for 1 day, to allow all larvae to reach the desired development stage at the end of P1. As discussed above, this ultimately did not result in significant differences between treatments, but T8 seemed to accelerate the development leading to bigger larvae in a more advanced development stage (Fig. 3A).

During BSFL P2, feed consumption was very similar between different treatments (Table S9). At the end of the trial, the percentage of remaining feed varied from 14.6 to 18.7% and larvae to feed mass ratios ranging from 0.233 to 0.260 were similar between treatments. At the end of P2, no significant differences were observed in terms of survival and yield, but the larvae of T8 again showed faster development, which can be observed from the larger number of (black) prepupal larvae (Fig. 3B). T3 showed the lowest number of prepupal larvae.

In the LMW trial, feed consumption and larvae to feed mass ratios were similar between treatments (Table S9). The calculated feed consumption varied between 9.4 and 16.7% and the larvae to feed mass ratio between 0.122 and 0.140.

### 3.3 Plant toxin levels and mass balance in BSFL and LMW

**Fig. 4:**
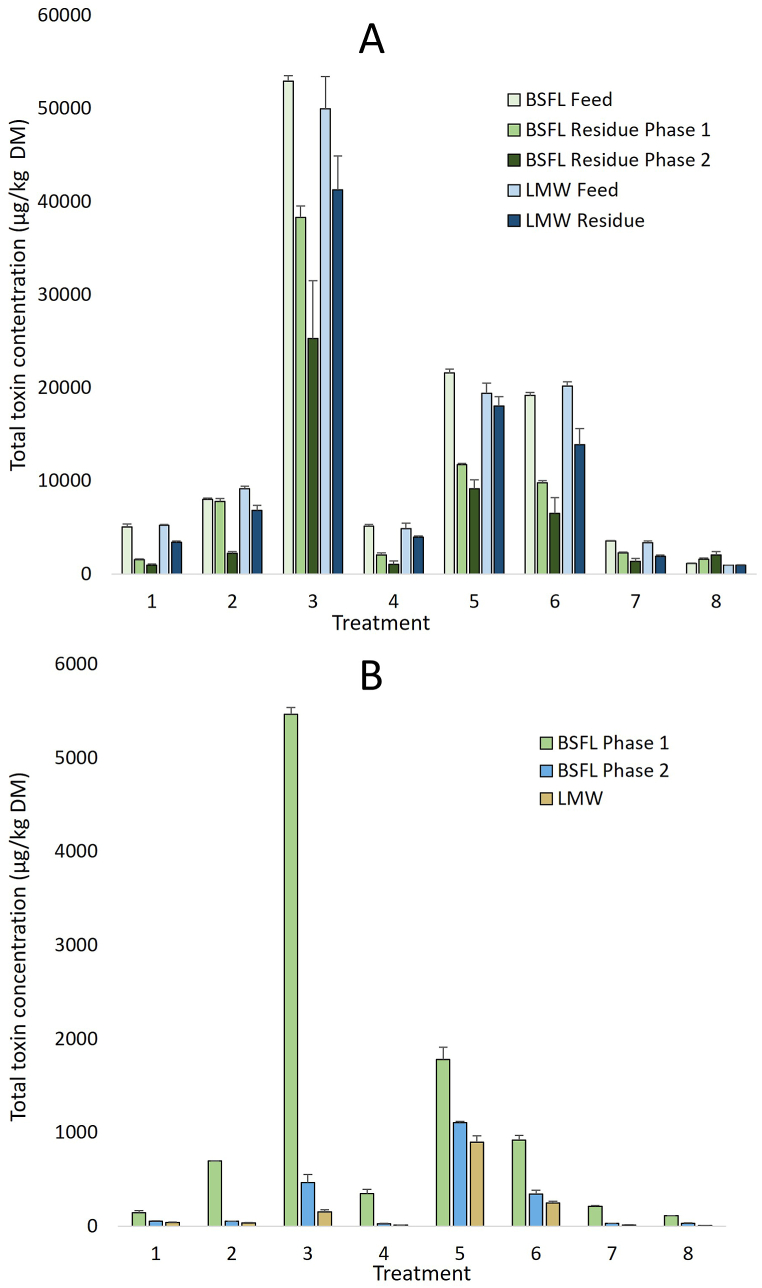
Total toxin concentration in BSFL and LMW starting feed and diet residues (μg/kg DM) (A) and total toxin concentrations in black soldier fly larvae (BSFL) and lesser mealworm (LMW) after harvesting (μg/kg DM) (B). Treatments: T1: Simulated J. vulgaris mix, T2: Simulated *S. vulgaris* mix, T3: Simulated S. inaequidens mix, T4: Simulated *E. vulgare* mix, T5: Simulated H. europaeum mix, T6: Simulated 5 herbs mix, T7: Atropine, T8: Scopolamine. Mean of N = 3 replicates, except for BSFL phase 1 (N = 2)*.*

The total toxin concentration in the residues was in general lower than that in the starting feeds (Fig. 4, Table S6), in line with the results obtained in the feed stability experiment. With respect to the recovery of toxins in the BSFL residue diets (Table S10), the largest reduction in total PA concentration was seen for T1. The total PA content declined from 5050 μg/kg to 1525 μg/kg (70% loss) in P1 residue and to 934 μg/kg (82% loss) in P2 residue. A relatively high recovery was observed for the feed in T2, for which only a 3% loss was observed in P1 residue. However, in P2 residue the total PA loss was 72%. In the other BSFL treatments, losses varying between 28% for P1 residue of T3 to 80% in P2 residue of T4 were observed. The reduction in concentration was also seen for atropine in T7 (up to 61% loss in BSFL P2 residue), but in contrast, the concentration of scopolamine in T8 increased by 38% in P1 residue and by 78% in P2 residue. This suggests that accumulation of scopolamine occurred in the residue of the BSFL experiments. For the LMW residues, the total PA content in general was closer to the total content in the starting feeds, although some recovery losses (7–35%) were observed here as well. A 43% reduction in the atropine content (T7) was observed, but the concentration of scopolamine (T8) remained unaffected.

As described in section 3.1, the composition of Pas in the feed substrates was not stable under the incubation conditions, particularly when the water content was high, as was the case in the BSFL trials. A substantial proportion of the PANOs was converted to the corresponding PAFBs and this is reflected in the measured composition in the residue (Table S6). In the BSFL P1 and P2 feeds, before addition of water, the proportion of PANOs varied between 66.5% in T1 and 93.4% in T5. In the residues remaining at the end of the trial, the proportion of PANOs was less than 1%. The situation was a bit different for the LMW experiments. Because fresh substrate was added every day to the containers, there was no complete conversion to PAFBs. The percentage PANOs in the residue varied from 38% in T1 to 67.7% in T5.

The concentrations of the toxins were generally higher in the BSFL P1 than in P2 larvae (Fig. 4B–Table S6). In P1 larvae the lowest concentrations were found in larvae grown in T1 with 142 μg Pa/kg DM and the highest in T3 with a total concentration of 5466 μg/kg DM. For BSFL P2, the lowest concentrations (24.6 μg/kg DM) were present in larvae raised in T4 and the highest concentrations (1104 μg/kg DM) were found in larvae from T5. The concentrations in LMW were generally lower than those in BSFL P2, but overall, they followed a similar trend. Lowest concentrations (9.9 μg/kg DM) were detected in T4 and highest concentrations (896 μg/kg DM) in T5. Atropine and scopolamine were also present in higher concentrations in BSFL P1 compared to P2 larvae. In LMW, concentrations were low, with atropine present at 14 μg/kg DM and scopolamine at 6.4 μg/kg DM.

PANOs were not detected in the BSFL, except for BSFL P1 in T5, in which PANOs contributed for 6.8% to the total PA content. The proportion of PANOs in the LMW varied from not detectable in T1 and T4 to 34% in T3. In the latter, retrorsine-NO, senecivernine-NO and usaramine-NO were detected in the larvae. Feed of T3 contained the highest PANO concentrations of the treatments used in this study, which may have facilitated the detection of PANOs in the larvae. The LOQ for individual Pas in larvae was 2.5 μg/kg DM and this, therefore, does not exclude the presence of low levels of PANOs in the larvae of other treatments.

### 3.4 Bioaccumulation and transfer factors of toxins in BSFL and LMW

**Fig. 5:**
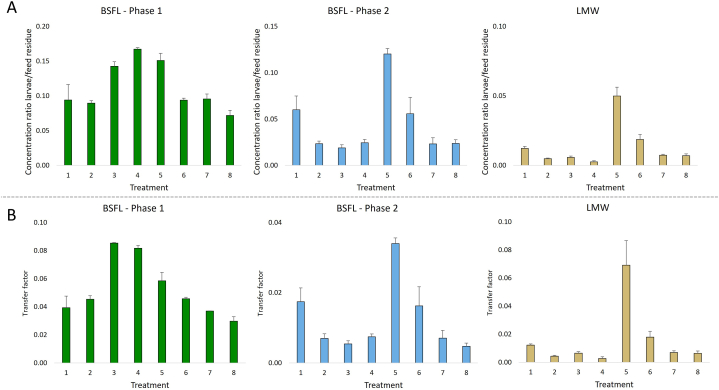
Bioaccumulation (5A) and transfer (5B) factors of plant toxins in black soldier fly larvae (BSFL) phase 1 and 2, and lesser mealworm (LMW) after harvest. Bioaccumulation factors are expressed as the ratio of the concentration in the larvae to that in the residue. Transfer factors are expressed as the amount of toxins in the larvae divided by the amount of toxins consumed from the residue. Treatments: T1: Simulated J. vulgaris mix, T2: Simulated *S. vulgaris* mix, T3: Simulated S. inaequidens mix, T4: Simulated *E. vulgare* mix, T5: Simulated H. europaeum mix, T6: Simulated 5 herbs mix, T7: Atropine, T8: Scopolamine. Mean of N = 3 replicates, except for BSFL phase 1 (N = 2).

The overall results for the BAFs are shown in Fig. 5A and data for individual toxins can be found in Table S11. All BAFs were <1, indicating that no bioaccumulation occurred. For BSFL P1, the BAFs varied between 0.07 (T8) and 0.17 (T4). Bioaccumulation factors for individual toxins (excluding PANOs) varied from 0.07 for lasiocarpine in T5 to 0.21 for sceleratine in T3 and rinderine in T6 (Table S11). In BSFL P2, in general the overall BAFs were lower, varying between 0.02 (T3) and 0.12 (T5). In T5, bioaccumulation in the larvae was clearly higher as compared to that of the other treatments. For individual toxins a wider range was seen in P2 compared to P1. The BAFs ranged from 0.01 to 0.18. Relatively high factors were observed for jacoline (0.18 ± 0.06, T1), jacobine (0.12 ± 0.05, T1), europine (0.16 ± 0.011, T5), rinderine (0.15 ± 0.01, T5) and echinatine (0.15 ± 0.05, T6). BAFs for the other Pas and the Tas atropine (0.02 ± 0.01) and scopolamine (0.02 ± 0.00) were below 0.1. In LMW the overall BAFs were low, ranging from 0.003 (T4) to 0.05 (T5). However, for individual toxins a wide range of factors was noted, varying from 0.002 to 0.53. Relatively high BAFs were found for europine (0.53 ± 0.10, T6), rinderine (0.25 ± 0.06, T6), echinatine (0.21 ± 0.04, T5) and jacoline (0.12 ± 0.02, T6). For the other compounds, including atropine (0.01 ± 0.00) and scopolamine (0.01 ± 0.00), BAFs were below 0.1.

The overall results on transfer of toxins to the larvae are presented in Fig. 5B and the results for individual toxins can be found in Table S12. Overall transfer factors (TFs) for BSFL P1 ranged from 0.030 (T8) to 0.084 (T3). TFs of the individual toxins ranged from 0.025 for retrorsine (T1) to 0.121 for sceleratine (T3). TFs for BSFL P2 were lower, ranging from 0.005 (T8) to 0.034 (T5). For individual toxins, the range was between 0.003 and 0.053. The highest TFs were found for jacoline (0.053 ± 0.016, T1), jacobine (0.036 ± 0.013, T1), europine (0.045 ± 0.003, T5), rinderine (0.041 ± 0.002) and echinatine (0.044 ± 0.014, T6). For the other toxins, including atropine (0.007 ± 0.002) and scopolamine (0.005 ± 0.001), TFs were below 0.03. The overall TFs for LMW ranged from 0.003 (T4) to 0.069 (T5). For individual compounds, the range was from 0.002 to 0.63. In LMW high TFs were calculated for europine (0.63 ± 0.17, T5), rinderine (0.32 ± 0.12, T5), echinatine (0.29 ± 0.09, T5) and jacoline (0.12 ± 0.02, T6). For the other compounds, including atropine (0.007 ± 0.001) and scopolamine (0.006 ± 0.001), TFs below 0.1 were calculated.

## 4 Discussion

### 4.1 Stability of toxins in feed materials and the consequences for transfer and bioaccumulation

The (in)stability of Pas in the substrates under the incubation conditions had a big impact on the PA composition and concentrations that the larvae were exposed to. Under wet or moist conditions, in the BSFL and LMW trial, respectively, the PANOs were readily converted to the corresponding PAFBs and/or degraded. There is a clear relationship with the dry matter content of the feeds: the higher the water content, the faster the conversion takes place. Degradation of PANOs has been reported before under silage and composting conditions [[32], [33], [34]]. In the silage, PANOs are not stable: a smaller proportion is reduced to the PAFBs, which thus may increase in concentration, and a larger part is degraded to unknown compounds [32]. The conversion is attributed to the microbiome present in the silage in combination with a high water content and a low pH. The conversion in our study is likely to be mediated by the microbiome present in the (non-sterile) feed, which is accelerated by favorable incubation conditions such as an elevated temperature and a high water content. The feed stability results strongly suggest that the conversion from PANOs to PAFBs is not mediated by the larvae. Nevertheless, some metabolism by the larvae cannot be excluded.

The conversion of PANOs in the insect feeds resulted in lower overall PA concentrations, but PAFB concentrations in general increased. The largest reduction in PA content was seen for treatment T1. In the residue of BSFL P1, only 30% was recovered and in the residual material of P2 this was only 18.5%. Even the PAFB content in these feed residues was lower than in the starting feed, although it should be remarked that the PAFB contribution in the starting material was relatively high (33.5%), as compared to the other treatments (6.6–21.4%). T1 contained Pas such as jacobine, jaconine and erucifoline and their N-oxides. Apparently, these PA analogues are less stable as compared to the PAFBs and PANOs in other treatments. Degradation of Pas present in *J. vulgaris* material during storage has been documented before (Crews et al., 2009). In T5 the strongest increase in PAFB content was seen (in BSFL P1 diet residue up to 725%), but this was also the treatment with the lowest amount of PAFBs in the starting feed (6.6%).

### 4.2 Transfer, bioaccumulation and metabolism of plant toxins in BSFL and LMW

It was noted that in BSFL P2 the amount of feed consumed (81–85%) was relatively high compared to the amount of feed consumed in P1 (33–53%). In the LMW trial the amount of feed consumed was relatively small, ranging from 9.4 to 16.7%. The diet residue, which is a mixture of non-consumed substrate, insect excrements (frass), remains of deceased larvae and sheddings, was analyzed at the end of each experiment. The mass balances obtained for the different treatments in the three experiments, expressed as the absolute amount of toxins recovered in the residue and the larvae, revealed that a significant amount of the Pas and Tas had disappeared at the end of each trial. With respect to the PANOs this disappearance can be largely attributed to the substrate microbiome, as was discussed above. However, the results Also indicate that, besides the conversion and degradation of PANOs by the substrate microbiome, the larvae themselves actively converted or degraded the PAFBs and TAs s. An important piece of evidence is that, except for scopolamine, the concentrations of the PAFBs and TAs in the residue of BSFL P2 were lower than those in the P1 residue. As stated above, the proportion of feed consumed in P2 was much larger than in P1. If no metabolism to novel compounds nor bioaccumulation in the larvae would take place, the levels in P2 residue would increase rather than decrease, as the toxins that are excreted via the frass would tend to concentrate in the residue that is left. Scopolamine was the only toxin that showed a clear concentration increase in the P2 residue compared to P1. Nevertheless, in absolute amounts partial degradation occurred for this compound as well. For the P1 residues, the overall recoveries in most treatments were also lower than those obtained in the feed stability experiment. This is an indication that also the P1 larvae actively convert or degrade the toxins and that excretion of intact PAs is a minor route. However, because the proportion of feed consumed in P1 was substantially smaller than in P2, definitive conclusions cannot be drawn. It cannot be excluded that microbial activity is higher in the feeds when the insects are present than when they are not. A difference with the stability test was that in the insect trials the feeds did not remain undisturbed but were kept in motion by the larvae. It is likely that the LMW also degraded the PAs that they consumed. However, in the LMW trials, only a small proportion of the feeds was consumed (9–17%), so this should have only a minor effect on the total amount of toxins present in the feeds.

The BSFL P1 showed a narrow range in bioaccumulation of plant toxins, with BAF ranging between 0.06 and 0.21. This suggests that P1 larvae did not actively discriminate between the different compounds, resulting in a relatively constant but also relatively high uptake. In contrast, a much wider variation in BAFs was seen in BSFL P2 and LMW, while at the same time the average factor values were lower, particularly for LMW. LMW treatment T5 and T6 are particularly interesting as some PA showed relatively high BAFs, such as europine (0.45–0.53), rinderine (0.23–0.25), echinatine (0.21) and to a lower extent heliotrine (0.042–0.049). These compounds are all PA monoesters. For PA diesters, such as echimidine (T4), lasiocarpine (T5) and heliosupine (T5), the bioaccumulation factors were small (BAF <0.01) or could not even be calculated, as for heliosupine. For the macrocyclic diesters the BAFs were also small in BSFL P2 and LMW, the only exceptions being jacoline and jacobine with BAFs above 0.1 in BSFL P2 and jacoline in LMW with a BAF of 0.072. For PANOs few BAFs could be calculated and only for a few treatments because the concentrations were very low. For BSFL P1, BAFs ranging between 2.2 and 6.4 were calculated for echimidine-NO, lasiocarpine-NO, europine-NO and heliotrine-NO; toxins that were present in T4 or T5. From the LMW trial, a larger number of BAFs could be calculated, because the PANO concentrations were higher in the residue feed. The factors all were very small, in the range of 0.002–0.009, which is even lower than those for the PAFBs in the LMW.

The values of the transfer factors were in line with the bioaccumulation factors. The TFs differed more strongly in the BSFL P2 compared to the P1 larvae, as was the case for the BAFs. The TFs for individual toxins in general were low, with values typically between 0.004 and 0.05 for BSFL P2 and LMW. Exceptions were europine (0.51–0.63), rinderine (0.24–0.32) and echinatine (0.29) in the LMW. These relatively high TFs indicate that these specific toxins were metabolized and/or excreted to a lesser amount than the other toxins.

The loss of the parent toxins in the overall mass balance, the low BAFs (with a few exceptions) and the low TFs (with a few exceptions) strongly suggests that the BSFL and LMW were able to convert the toxins to novel substances. Prior research on mycotoxins in relation to reared insects has suggested that emptying the digestive tract by fasting, or providing ‘clean’ feed for a period of time before harvest, could reduce respective mycotoxin concentrations in the final larval product [35] – although there are some concerns over ‘insect welfare’ of this practice [36]. It is recommended that any such effects of a fasting regimen are studied further for the plant toxins identified in this study. The structure of these metabolic breakdown products was not further investigated, and it would require a separate study to address this in sufficient detail. However, as the PAs and TAs contain ester groups, it can be hypothesized that hydrolysis of these esters may be one of the degradation routes. Hydrolysis would result in the formation of necine bases and necic acids in case of PAs and to tropine and tropic acid in case of atropine and scopolamine. Previous studies with milking cows and laying hens – in addition to extensive degradation of PAs – showed the formation of new metabolites [19,37]. These new metabolites, often hydroxylated versions of the PAs present in the plant material, were not detected in the current study.

### 4.3 Animal and human health perspectives

The toxin content of the treatments realistically simulated the composition encountered in various herbs. The limited number of biological replicates per treatment in this study imply some statistical limitations of the conclusions in terms of insect performance. More research is thus recommended to validate these findings at larger scale and with more repetitions. None of the treatments appeared to result in an increased insect mortality. A temporary impairment of growth and a lower feed consumption were observed for BSFL P1 raised on T3, that simulated the presence of *S. inaequidens* at an inclusion level of 1% to the feed. This feed contained the highest total amount of PAs (53 mg/kg DM) of all treatments tested. In the second phase of the experiment, no growth impairment was observed, indicating that the BSFL at that stage were more resistant to relatively high PA levels in their feed. The higher overall toxin concentrations in BSFL P1 compared to P2 may also provide an indication that at an early development stage the larvae have more difficulty to cope with the toxins while at a later development stage they are better adapted and capable to reduce the levels, e.g., by an increased excretion or metabolism. For LMW at the end of the trial, growth impairment was not observed, although this does not fully exclude the possibility of a lower growth rate in the early stages of the trial. BSFL and LMW are generalist herbivores that are not specifically adapted to PAs or TAs and, therefore, can be expected to have a limited capacity in coping with these toxic plant metabolites. Increased mortality as well as feeding preferences have been reported for several insect herbivores in feeding trials and choice tests that involved PAs [[38], [39], [40]]. However, these tests have been conducted with pure substances, plants or plant materials, whereas in our study the concentrations to which the larvae were exposed were substantially lower.

From a human food safety perspective, it must first be highlighted that if insects reared for food and feed purposes are produced in compliance with hygienic standards such as HACCP, the insect-based products can be considered safe for food and feed. However, accidental contamination of insect substrates with a variety of plant toxins originating from herbs are a potential risk to be considered. PAs are primarily of concern because of their potential genotoxic carcinogenic properties (EFSA, 2011). In 2017 EFSA concluded that an exposure of 0.0237 μg PA/kg BW per day, corresponding to 1.66 μg per day for a 70-kg person, would be of low concern [41]. In our study, the uptake by LMW varied between 33 μg/kg DM (treatment T2) and 832 μg/kg DM (treatment T5). Assuming a serving of 30 g larvae (corresponding to approximately 10 g on a DM basis), this would represent a human intake of 0.3–8.3 μg PA. For the LMW with the highest PA content this would result in an exceedance of the level of low concern by a factor 5. However, considering that the inclusion of insects in human diets is rather limited, we conclude that based on the observed levels in the current study, the intake via the consumption of insects accidently contaminated with PAs would be very small.

The same can be concluded for the TAs. Atropine and scopolamine are anticholinergic agents that block muscarinic acetylcholine receptors. They affect the peripheral nervous system (PNS) as well as the central nervous system (CNS) and exert effects on the heart rate and blood pressure [42]. EFSA established an Acute Reference Dose (ARfD) of 0.016 μg/kg BW for the sum of (−)-hyoscyamine and (−)-scopolamine, assuming equivalent potency. For an adult of 70 kg this corresponds to an intake of approx. 1 μg. The concentrations of atropine and scopolamine found in LMW were quite low, 14.0 and 6.4 μg/kg DM, respectively. A serving of 30 g larvae would therefore result in an intake of TAs well below the ARfD and thus would be of no concern.

Finally, as an alternative to the inclusion of insects reared on substrates contaminated with plant toxins in food or feed products, other bioremediation outcomes could be considered [43]. As discussed above; although the results from this study do not immediately cause concern for the presence of low concentrations of plant toxins in the substrates of tested species, higher concentrations may yield different results. Further, given the potential genotoxicity of PAs, application of the precautionary principle may preclude the commercialization within the food chain of any exposed insects. Alternative ‘entomoremediation’ options could, for instance, include use as a bio-fuel, fertilizer, or a variety of high-value chitin-based industrial applications [44,45].

## 5 Conclusion

The quality of the insect feed not only can have implications for the growth and development of the insects, but it also has food safety aspects. In this study, we investigated the transfer and bioaccumulation of two specific groups of plant toxins, pyrrolizidine and tropane alkaloids, secondary metabolites that are produced by a wide variety of plants as defence compounds against predators. BSFL and LMW were grown on a set of 8 different substrates that differed in toxin composition and concentration. A temporary impairment of growth was only noted for BSFL P1 that were raised on the treatment that simulated the presence of *S. inaequidens* PAs at an inclusion level of 1% to the feed. This feed contained the highest total amount of PAs of all treatments tested. In the second phase of the experiment, no growth impairment was observed, indicating that the BSFL were better capable to cope with relatively high levels of PAs in their feed. For LMW at the end of the trial no growth impairment was noted as well.

In the substrates, PA N-oxides were quickly converted to the corresponding tertiary amines and partial degradation of PAs occurred as well. This finding shows that it is important to check the stability of compounds under the experimental conditions used in the insect tests.

Both BSFL and LMW seem capable of degrading the plant toxins they were exposed to. Newly formed metabolites were not detected in any of the treatments, suggesting that this is not an important route of detoxification for the larvae, although follow-up research to determine the identify of further degradation products is recommended. Values for bioaccumulation and transfer varied widely; highest values were observed for the PA monoesters europine, echinatine, rinderine and heliotrine. These PAs accumulated more strongly in LMW than in BSFL. For most other PAs as well as the TAs atropine and scopolamine bioaccumulation factors were very low. Overall, plant toxin concentrations found in insects were low.

## Funding sources

This project has received funding from the European Union's 10.13039/501100007601Horizon 2020 Research and Innovation Programme under grant agreement No 861976 (Project SUSINCHAIN). Additional financing from the Netherlands Ministry of Agriculture, Nature and Food Quality (Knowledge base program KB34, project BO-57-102-005) is acknowledged.

## Data availability statement

Data included in article/supp. material/referenced in article.

## CRediT authorship contribution statement

**Patrick P.J. Mulder:** Writing – original draft, Visualization, Formal analysis, Conceptualization. **Judith T.L. Mueller-Maatsch:** Writing – review & editing, Methodology, Investigation, Formal analysis, Conceptualization. **Nathan Meijer:** Writing – original draft, Visualization, Conceptualization. **Marlou Bosch:** Writing – review & editing, Investigation, Conceptualization. **Lisa Zoet:** Writing – review & editing, Visualization, Investigation, Conceptualization. **H.J. Van Der Fels-Klerx:** Writing – review & editing, Supervision, Resources, Project administration, Funding acquisition, Conceptualization.

## Declaration of competing interest

The authors declare the following financial interests/personal relationships which may be considered as potential competing interests: This project has received funding from the European Union's 10.13039/501100007601Horizon 2020 research and innovation programme under grant agreement No 861976 (project SUSINCHAIN). Additional financing from the Netherlands Ministry of Agriculture, Nature and Food Quality (through project BO-57-102-005) is acknowledged. Co-author Lisa Zoet reports a relationship with Bestico BV that includes employment. Bestico B.V. is a commercial entity producing black soldier fly larvae for animal feed purposes. Other authors declare that they have no known competing financial interests or personal relationships that could have appeared to influence the work reported in this paper.
