# Supplementary material for: Effects of dietary exposure to plant toxins on bioaccumulation, survival, and growth of black soldier fly (Hermetia illucens) larvae and lesser mealworm (Alphitobius diaperinus)
Source: Heliyon. 2024 Feb 16;10(4):e26523. doi: 10.1016/j.heliyon.2024.e26523 (PMC10884485; doi:10.1016/j.heliyon.2024.e26523)
Supplement: Multimedia component 1 [file mmc1.docx]

Effects of dietary exposure to plant toxins on bioaccumulation, survival, and growth of black soldier fly (*Hermetia illucens*) larvae and lesser mealworm (*Alphitobius diaperinus*)

P.P.J. Mulder^1^, J.T.L. Mueller-Maatsch^1^, N. Meijer^1*^, M. Bosch^2^, L. Zoet^3^, H.J. van der Fels-Klerx^1^

^1^ Wageningen Food Safety Research (WFSR), part of Wageningen University and Research, Akkermaalsbos 2, 6708 WB Wageningen, The Netherlands

^2^ Ynsect NL (formerly Protifarm), Harderwijkerweg 141a, 3852 AB Ermelo, The Netherlands

^3^ Bestico, Industrieweg 6, 2651 BE Berkel en Rodenrijs, the Netherlands

* Corresponding author:

Nathan Meijer

E-Mail: Nathan.meijer@wur.nl

Telephone: +31(0)614615684

**Supplementary Material**

**Table S1:** PA and TA standards used in this study. Details on vendors and purity.

| **Pyrrolizidine/tropane alkaloid** | **CAS no.** | **Supplier** | **Article code** | **Purity (%)** |
| --- | --- | --- | --- | --- |
| Atropine | 51-55-8 | Sigma-Aldrich | A0132 | >99 |
| Atropine-d3 (IS) |  | CDN Isotopes | D-1861 | 99.4 |
| Dibutylheliotridine-d2 (IS) |  | ChiroBlock | UWA 4577 | 96.2 |
| Dibutylheliotridine N-oxide-d2 (IS) |  | ChiroBlock | UWA 4577 | 95.2 |
| Echimidine | 520-68-3 | PhytoPlan | 6278.95 | 97.78 |
| Echimidine N-oxide | 41093-89-4 | PhytoPlan | 6279.97 | >97 |
| Echinatine | 480-83-1 | PhytoPlan | 6295.95 | >95 |
| Echinatine N-oxide | 202067-93-0 | PhytoPlan | 6296.95 | >95 |
| Epi-jacobine (IS) |  | Mercachem | ME20060158 | >95 |
| Epi-jacobine N-oxide (IS) |  | Mercachem | ME20060158 | >95 |
| Erucifoline | 40158-95-0 | PhytoPlan | 6218.97 | 99.91 |
| Erucifoline N-oxide | 123864-94-0 | PhytoPlan | 6221.97 | 99.6 |
| Europine (hydrochloride) | 570-19-4 | PhytoPlan | 6214.97 | >97 |
| Europine N-oxide | 65582-53-8 | PhytoPlan | 6215.97 | 99.73 |
| Heliosupine | 32728-78-2 | PhytoPlan | 6297.95 | >95 |
| Heliosupine N-oxide | 31701-88-9 | PhytoPlan | 6297.95 | >95 |
| Heliotrine | 303-33-3 | PhytoPlan | 6212.98 | 98.96 |
| Heliotrine N-oxide | 6209-65-0 | PhytoPlan | 6213.97 | >97 |
| Indicine (hydrochloride) | 1195140-94-3 | PhytoLab | 83234 | 98.9 |
| Indicine N-oxide | 41708-76-3 | PhytoPlan | 6217.96 | >96 |
| Integerrimine | 480-79-5 | PhytoPlan | 6283.97 | >97 |
| Integerrimine N-oxide | 85955-28-8 | PhytoPlan | 6284.95 | >95 |
| Intermedine | 10285-06-0 | PhytoPlan | 6274.95 | >95 |
| Intermedine N-oxide | 95462-14-9 | PhytoPlan | 6275.95 | >95 |
| Jacobine | 6870-67-3 | PhytoPlan | 6219.98 | >98 |
| Jacobine N-oxide | 38710-25-7 | PhytoPlan | 6222.96 | 96.45 |
| Jacoline | 480-76-2 | PhytoPlan | 6291.97 | >97 |
| Jacoline N-oxide | 1148039-73-9 | PhytoPlan | 6292.97 | >97 |
| Jaconine | 480-75-1 | PhytoPlan | 6293.95 | >95 |
| Jaconine N-oxide | 1148039-75-1 | PhytoPlan | 6294.95 | >95 |
| Lasiocarpine | 303-34-4 | PhytoPlan | 6210.97 | >97 |
| Lasiocarpine N-oxide | 127-30-0 | PhytoPlan | 6211.96 | 96.74 |
| Lycopsamine | 10285-07-1 | PhytoPlan | 6270.95 | 97.45 |
| Lycopsamine-d7 (IS) |  | TRC | L487527 | 99.3 |
| Lycopsamine N-oxide | 95462-15-0 | PhytoPlan | 6271.95 | 98.74 |
| Lycopsamine N-oxide-d7 (IS) |  | TRC | L487532 | 99.2 |
| Retrorsine | 480-54-6 | PhytoPlan | 6203.98 | >98 |
| Retrorsine N-oxide | 15503-86-3 | PhytoPlan | 6253.96 | >96 |
| Riddelliine | 23246-96-0 | PhytoPlan | 6312.98 | >98 |
| Riddelliine N-oxide | 75056-11-0 | PhytoPlan | 6313.97 | >97 |
| Rinderine | 6029-84-1 | PhytoPlan | 6310.95 | >95 |
| Rinderine N-oxide | 137821-16-0 | PhytoPlan | 6311.95 | >95 |
| Sceleratine | 6190-26-6 | PhytoPlan | 6229.97 | >97 |
| Sceleratine N-oxide | 103184-92-5 | PhytoPlan | 6230.97 | >97 |
| Scopolamine (hydrobromide trihydrate) | 6533-68-2 | Sigma Aldrich | S1875 | >98 |
| Scopolamine-d3 (hydrobromide trihydrate) (IS) |  | CDN Isotopes | D-3815 | 99.5 |
| Senecionine | 130-01-8 | PhytoPlan | 6202.99 | >99 |
| Senecionine-d3 (IS) |  | TRC | S258652 | 98.9 |
| Senecionine N-oxide | 13268-67-2 | PhytoPlan | 6252.95 | >95 |
| Senecionine N-oxide-d3 (IS) |  | TRC | S258657 | 99.1 |
| Seneciphylline | 480-81-9 | PhytoLab | 89275 | 99.8 |
| Seneciphylline N-oxide | 38710-26-8 | PhytoLab | 82632 | 99.6 |
| Senecivernine | 72755-25-0 | PhytoPlan | 6206.95 | 98.95 |
| Senecivernine N-oxide | 101687-28-9 | PhytoPlan | 6220.95 | 99.47 |
| Senkirkine | 2318-18-5 | PhytoPlan | 6205.95 | 98.01 |
| Spartioidine | 520-59-2 | PhytoPlan | 6314.95 | 95.1 |
| Spartioidine N-oxide | 121123-61-3 | Phytoplan | 6323.95 | 97.9 |
| Usaramine | 15503-87-4 | PhytoPlan | 6315.96 | >96 |
| Usaramine N-oxide | 117020-54-9 | PhytoPlan | 6316.96 | >96 |

**Table S2:** Preparation of spiked materials 1 to 6. Levels are based on the concentrations determined in feed materials described in Mulder et al, 2016.

| **Material 1: Simulated *Jacobaea vulgaris*** | | |  | **Material 2: Simulated *Senecio vulgaris*** | | |  | **Material 3: Simulated *Senecio inaequidens*** | | |
| --- | --- | --- | --- | --- | --- | --- | --- | --- | --- | --- |
| **Pyrrolizidine alkaloid** | **Feed ^a^** | **Feed ^b,c^** |  | **Pyrrolizidine alkaloid** | **Feed ^a^** | **Feed ^b^** |  | **Pyrrolizidine alkaloid** | **Feed ^a^** | **Feed ^b^** |
|  | **(µg/kg)** | **(µg/kg)** |  |  | **(µg/kg)** | **(µg/kg)** |  |  | **(µg/kg)** | **(µg/kg)** |
| Erucifoline | 550 | 550 |  | Integerrimine | 50 | 50 |  | Integerrimine | 180 | 180 |
| Erucifoline N-oxide | 1160 | 1160 |  | Integerrimine N-oxide | 710 | 710 |  | Integerrimine N-oxide | 1670 | 1670 |
| Integerrimine | 30 | 30 |  | Retrorsine | 130 | 130 |  | Retrorsine | 2500 | 2500 |
| Integerrimine N-oxide | 240 | 240 |  | Retrorsine N-oxide | 1720 | 1720 |  | Retrorsine N-oxide | 23800 | 23800 |
| Jacobine | 260 | 260 |  | Riddelliine | 30 | 30 |  | Sceleratine | 130 | 130 |
| Jacobine N-oxide | 870 | 870 |  | Riddelliine N-oxide | 270 | 270 |  | Sceleratine N-oxide | 340 | 340 |
| Jacoline | 80 | 80 |  | Senecionine | 170 | 170 |  | Senecionine | 310 | 310 |
| Jacoline N-oxide | 120 | 120 |  | Senecionine N-oxide | 1660 | 1660 |  | Senecionine N-oxide | 5000 | 5000 |
| Jaconine | 20 | 20 |  | Seneciphylline | 620 | 620 |  | Senecivernine | 870 | 870 |
| Retrorsine | 40 | 40 |  | Seneciphylline N-oxide | 4900 | 4900 |  | Senecivernine N-oxide | 8700 | 8700 |
| Retrorsine N-oxide | 170 | 170 |  | Senecivernine | 20 | 20 |  | Senkirkine | 30 | 6650 ^e^ |
| Riddelliine | 10 | - |  | Senecivernine N-oxide | 150 | 150 |  | Usaramine | 330 | 330 |
| Riddelliine N-oxide | 40 | 40 |  | Spartioidine | 90 | 90 |  | Usaramine N-oxide | 4100 | 4100 |
| Senecionine | 190 | 190 |  | Spartioidine N-oxide | 540 | - ^d^ |  | Desacetyldoronine* | 10 | - |
| Senecionine N-oxide | 840 | 840 |  | Usaramine N-oxide | 20 | 20 |  | Doronine* | 10 | - |
| Seneciphylline | 170 | 170 |  | Cyclic diester 338* | 20 | - |  | Floridanine* | 450 | - |
| Seneciphylline N-oxide | 620 | 620 |  | Cyclic diester N-oxide 366* | 40 | - |  | Florosenine* | 2100 | - |
| Spartioidine | 10 | - |  |  |  |  |  | Onetine* | 620 | - |
| Spartoidine N-oxide | 10 | - |  |  |  |  |  | Otosenine* | 2100 | - |
| Usaramine N-oxide | 30 | 30 |  |  |  |  |  | Macrocyclic diester 334* | 10 | - |
| Acetylerucifoline N-oxide* | 20 | - |  |  |  |  |  | Cyclic diester N-oxide 350* | 70 | - |
| Hydroxyjacobine* | 10 | - |  |  |  |  |  | Cyclic diester N-oxide 366* | 230 | - |
| Jacozine* | 10 | - |  |  |  |  |  |  |  |  |
| Jacozine N-oxide* | 30 | - |  |  |  |  |  |  |  |  |
|  |  |  |  |  |  |  |  |  |  |  |
| Total (µg/kg) | 5530 | 5430 |  |  | 11080 | 10540 |  |  | 53560 | 47930 |
|  |  |  |  |  |  |  |  |  |  |  |
| % Composition covered |  | 98.2% |  |  |  | 95.1% |  |  |  | 89.5% |
|  |  |  |  |  |  |  |  |  |  |  |
| * No commercial standard available or compound with undefined chemical structure | | | | | | | | | | |
| a Composition according to Mulder et al, 2016 | | | | | | | | | | |
| b Artificial feed produced | | | | | | | | | | |
| c PAs present at low level (<20 ug/kg) were not spiked | | | | | | | | | | |
| d Spartioidine N-oxide was not available in sufficient amounts for spiking | | | | | | | | | | |
| e Senkirkine was added in excess to mimick the otonecine PAs present in *S. inaequidens* | | | | | | | | | | |

| **Material 4: Simulated *Echium vulgare*** | | |  | **Material 5: Simulated *Heliotropium europaeum*** | | |  | **Material 6: Five herbs mix** | |
| --- | --- | --- | --- | --- | --- | --- | --- | --- | --- |
| **Pyrrolizidine alkaloid** | **Feed ^a^** | **Feed ^b,c^** |  | **Pyrrolizidine alkaloid** | **Feed ^a^** | **Feed ^b,c^** |  | **Pyrrolizidine alkaloid** | **Feed ^b,d^** |
|  | **(µg/kg)** | **(µg/kg)** |  |  | **(µg/kg)** | **(µg/kg)** |  |  | **(µg/kg)** |
| Echimidine | 370 | 370 |  | Echinatine N-oxide | 130 | 130 |  | Echimidine | 74 |
| Echimidine N-oxide | 5100 | 5100 |  | Europine | 230 | 230 |  | Echimidine N-oxide | 1020 |
| Lycopsamine N-oxide | 10 | - |  | Europine N-oxide | 5800 | 5800 |  | Echinatine N-oxide | 26 |
| 3'-Acetylechimidine N-oxide* | 10 | - |  | Heliosupine | 30 | 30 |  | Erucifoline | 110 |
| Open diester 398* | 40 | - |  | Heliosupine N-oxide | 170 | 170 |  | Erucifoline N-oxide | 232 |
| Open diester N-oxide 414* | 430 | - |  | Heliotrine | 420 | 420 |  | Europine | 46 |
|  |  |  |  | Heliotrine N-oxide | 8000 | 8000 |  | Europine N-oxide | 1160 |
|  |  |  |  | Intermedine N-oxide | 10 | - |  | Heliosupine | 6 |
|  |  |  |  | Lasiocarpine | 270 | 270 |  | Heliosupine N-oxide | 34 |
|  |  |  |  | Lasiocarpine N-oxide | 4300 | 4300 |  | Heliotrine | 84 |
|  |  |  |  | Rinderine | 10 | - |  | Heliotrine N-oxide | 1600 |
|  |  |  |  | Rinderine N-oxide | 320 | 320 |  | Integerrimine | 78 |
|  |  |  |  | 5'-Acetyleuropine N-oxide* | 140 | - |  | Integerrimine N-oxide | 1190 |
|  |  |  |  | 3'-Acetylheliosupine N-oxide* | 30 | - |  | Jacobine | 52 |
|  |  |  |  | 5'-Acetyllasiocarpine N-oxide* | 560 | - |  | Jacobine N-oxide | 174 |
|  |  |  |  | Heulerine* | 50 | - |  | Jacoline | 16 |
|  |  |  |  | Heulerine N-oxide* | 940 | - |  | Jacoline N-oxide | 24 |
|  |  |  |  | *Iso*-Lasiocarpine N-oxide* | 40 | - |  | Jaconine | 4 |
|  |  |  |  | Supinine* | 10 | - |  | Lasiocarpine | 54 |
|  |  |  |  | Supinine N-oxide* | 40 | - |  | Lasiocarpine N-oxide | 860 |
|  |  |  |  |  |  |  |  | Retrorsine | 534 |
|  |  |  |  |  |  |  |  | Retrorsine N-oxide | 5138 |
|  |  |  |  |  |  |  |  | Riddelliine | 6 |
|  |  |  |  |  |  |  |  | Riddelliine N-oxide | 62 |
|  |  |  |  |  |  |  |  | Rinderine N-oxide | 64 |
|  |  |  |  |  |  |  |  | Sceleratine | 26 |
|  |  |  |  |  |  |  |  | Sceleratine N-oxide | 68 |
|  |  |  |  |  |  |  |  | Senecionine | 108 |
|  |  |  |  |  |  |  |  | Senecionine N-oxide | 834 |
|  |  |  |  |  |  |  |  | Seneciphylline | 158 |
|  |  |  |  |  |  |  |  | Seneciphylline N-oxide | 1104 |
|  |  |  |  |  |  |  |  | Senecivernine | 178 |
|  |  |  |  |  |  |  |  | Senecivernine N-oxide | 1770 |
|  |  |  |  |  |  |  |  | Senkirkine | 1330 |
|  |  |  |  |  |  |  |  | Spartioidine | 18 |
|  |  |  |  |  |  |  |  | Usaramine | 66 |
|  |  |  |  |  |  |  |  | Usaramine N-oxide | 830 |
|  |  |  |  |  |  |  |  |  |  |
| Total (µg/kg) | 5960 | 5470 |  |  | 21500 | 19670 |  |  | 19138 |
| % Composition covered |  | 91.8% |  |  |  | 91.5% |  |  |  |
|  |  |  |  |  |  |  |  |  |  |
| * No commercial standard available or compound with undefined chemical structure | | | | | | | | | |
| a Composition according to Mulder et al, 2016 | | | | | | | | | |
| b Artificial feed produced | | | | | | | | | |
| c PAs present at low level (<20 ug/kg) were not spiked | | | | | | | | | |
| d Composition based on the relative contributions of materials 1-5 (20% each) | | | | | | | | | |

**Table S3:** Dry matter (DM) content of BSF and LMW starting feed materials, diet residue and larvae.

|  | **BSF starting feed** | **BSF**  **feed stability test, set 1** | **BSF**  **feed stability test, set 2** | **BSF**  **diet residue phase 1** | **BSF**  **diet residue phase 2** | **BSF larvae phase 1** | **BSF larvae phase 2** | **LMW starting feed** | **LMW**  **feed stability test** | **LMW**  **diet residue** | **LMW larvae** |
| --- | --- | --- | --- | --- | --- | --- | --- | --- | --- | --- | --- |
| N | 9 | 9 | 9 | 18 | 27 | 18 | 27 | 9 | 18 | 27 | 27 |
| Avg. DM content (%) | 92.2% | 50.5% | 32.7% | 58.9% | 31.2% | 30.1% | 31.1% | 91.3% | 87.0% | 85.0% | 31.6% |
| SD (%) | 0.7% | 4.5% | 0.6% | 3.9% | 2.5% | 1.4% | 1.4% | 0.4% | 1.7% | 1.3% | 1.2% |
| RSD (%) | 0.8% | 9.0% | 1.9% | 6.7% | 7.9% | 4.7% | 4.7% | 0.5% | 1.9% | 1.5% | 3.7% |

**Table S4:** Mass spectrometric settings used for the analysis of pyrrolizidine and tropane alkaloids.

| **Pyrrolizidine/tropane alkaloid** | **Precursor ion**  **(m/z)** | **Cone voltage (V)** | **Product ion 1 (m/z)** | **Col. energy 1 (eV)** | **Product ion 2 (m/z)** | **Col. energy 2 (eV)** | **Product ion 3 (m/z)** | **Col. energy 3 (eV)** | **Indicative RT**  **(min)** | **MRM window** | **IS used** |
| --- | --- | --- | --- | --- | --- | --- | --- | --- | --- | --- | --- |
| Atropine | 290.2 | 30 | 93 | 20 | 124 | 30 |  |  | 7.85 | 4 | IS-1 |
| Atropine-d3 (IS-1) | 293.2 | 30 | 93 | 20 | 127 | 30 |  |  | 7.75 | 4 |  |
| Dibutylheliotridine-d2 (IS-2) | 298.2 | 30 | 122 | 40 | 140 | 35 |  |  | 11.85 | 6 |  |
| Dibutylheliotridine N-oxide-d2 (IS-3) | 314.2 | 30 | 120 | 30 | 138 | 35 |  |  | 9.05 | 4 |  |
| Echimidine | 398.2 | 30 | 120 | 25 | 220 | 20 | 83 | 25 | 10.55 | 6 | IS-2 |
| Echimidine N-oxide | 414.2 | 30 | 254 | 30 | 352 | 25 | 94 | 40 | 7.40 | 4 | IS-3 |
| Echinatine | 300.2 | 30 | 138 | 30 | 156 | 30 | 94 | 35 | 6.45 | 4 | IS-6 |
| Echinatine N-oxide | 316.2 | 30 | 111 | 40 | 172 | 30 | 94 | 40 | 3.75 | 5 | IS-7 |
| Epi-jacobine (IS-4) | 352.2 | 40 | 120 | 30 | 155 | 30 | 94 | 40 | 8.85 | 1 |  |
| Epi-jacobine N-oxide (IS-5) | 368.2 | 40 | 120 | 30 | 296 | 25 | 119 | 30 | 5.75 | 2 |  |
| Erucifoline | 350.2 | 40 | 94 | 40 | 120 | 30 | 138 | 30 | 7.30 | 1 | IS-4 |
| Erucifoline N-oxide | 366.2 | 40 | 94 | 40 | 118 | 30 | 120 | 30 | 3.25 | 2 | IS-5 |
| Europine | 330.2 | 30 | 94 | 35 | 138 | 30 |  |  | 6.15 | 5 | IS-6 |
| Europine N-oxide | 346.2 | 30 | 172 | 30 | 111 | 40 |  |  | 3.55 | 5 | IS-7 |
| Heliosupine | 398.2 | 30 | 120 | 25 | 220 | 20 | 336 | 20 | 10.40 | 6 | IS-2 |
| Heliosupine N-oxide | 414.2 | 30 | 94 | 30 | 254 | 30 | 138 | 30 | 7.10 | 4 | IS-3 |
| Heliotrine | 314.2 | 30 | 138 | 25 | 156 | 25 |  |  | 8.10 | 4 | IS-6 |
| Heliotrine N-oxide | 330.2 | 30 | 172 | 25 | 94 | 40 |  |  | 5.50 | 5 | IS-7 |
| Indicine | 300.2 | 30 | 94 | 35 | 156 | 30 | 138 | 30 | 5.50 ^a^ | 4 | IS-6 |
| Indicine N-oxide | 316.2 | 30 | 94 | 40 | 172 | 30 | 111 | 40 | 3.55 | 5 | IS-7 |
| Integerrimine | 336.2 | 40 | 94 | 40 | 120 | 30 | 138 | 30 | 9.95 | 3 | IS-9 |
| Integerrimine N-oxide | 352.2 | 40 | 94 | 40 | 120 | 30 | 136 | 30 | 6.50 | 1 | IS-10 |
| Intermedine | 300.2 | 30 | 94 | 35 | 156 | 30 | 138 | 30 | 5.45 ^a^ | 4 | IS-6 |
| Intermedine N-oxide | 316.2 | 30 | 94 | 40 | 172 | 30 | 111 | 40 | 3.40 ^b^ | 5 | IS-7 |
| Jacobine | 352.2 | 40 | 120 | 30 | 155 | 30 | 94 | 40 | 7.65 | 1 | IS-4 |
| Jacobine N-oxide | 368.2 | 40 | 120 | 30 | 296 | 25 | 119 | 30 | 4.40 | 2 | IS-5 |
| Jacoline | 370.2 | 40 | 94 | 40 | 138 | 30 |  |  | 5.35 | 2 | IS-4 |
| Jacoline N-oxide | 386.2 | 40 | 94 | 40 | 120 | 30 |  |  | 2.60 | 1 | IS-5 |
| Jaconine | 388.2 | 40 | 94 | 40 | 120 | 30 |  |  | 8.75 | 3 | IS-4 |
| Jaconine N-oxide | 404.2 | 40 | 94 | 40 | 120 | 35 |  |  | 4.95 | 1 | IS-5 |
| Lasiocarpine | 412.2 | 30 | 120 | 50 | 220 | 25 |  |  | 11.25 | 6 | IS-2 |
| Lasiocarpine N-oxide | 428.2 | 30 | 138 | 30 | 254 | 25 |  |  | 8.25 | 6 | IS-3 |
| Lycopsamine | 300.2 | 30 | 94 | 35 | 156 | 30 | 138 | 30 | 5.50 ^a^ | 4 | IS-6 |
| Lycopsamine-d7 (IS-6) | 307.2 | 30 | 94 | 35 | 156 | 30 |  |  | 5.45 | 4 |  |
| Lycopsamine N-oxide | 316.2 | 30 | 94 | 40 | 172 | 30 | 111 | 40 | 3.45 ^b^ | 5 | IS-7 |
| Lycopsamine N-oxide-d7 (IS-7) | 323.2 | 30 | 94 | 40 | 172 | 30 |  |  | 3.40 | 5 |  |
| Retrorsine | 352.2 | 40 | 94 | 40 | 120 | 30 | 138 | 30 | 8.55 | 1 | IS-4 |
| Retrorsine N-oxide | 368.2 | 40 | 94 | 40 | 120 | 30 | 119 | 30 | 5.40 | 2 | IS-5 |
| Riddelliine | 350.2 | 40 | 94 | 40 | 120 | 30 | 138 | 30 | 7.80 | 1 | IS-4 |
| Riddelliine N-oxide | 366.2 | 40 | 94 | 40 | 118 | 30 | 120 | 30 | 4.50 | 2 | IS-5 |
| Rinderine | 300.2 | 30 | 138 | 30 | 156 | 30 | 94 | 35 | 6.60 | 4 | IS-6 |
| Rinderine N-oxide | 316.2 | 30 | 111 | 40 | 172 | 30 | 94 | 40 | 3.85 | 5 | IS-7 |
| Sceleratine | 370.2 | 40 | 94 | 40 | 138 | 30 | 120 | 30 | 5.75 | 2 | IS-4 |
| Sceleratine N-oxide | 386.2 | 40 | 94 | 40 | 120 | 30 |  |  | 2.80 | 1 | IS-5 |
| Scopolamine | 304.2 | 30 | 138 | 15 | 156 | 25 |  |  | 8.45 | 4 | IS-8 |
| Scopolamine-d3 (IS-8) | 307.2 | 30 | 141 | 15 | 156 | 25 |  |  | 8.40 | 4 |  |
| Senecionine | 336.2 | 40 | 94 | 40 | 120 | 30 | 138 | 30 | 10.25 | 3 | IS-9 |
| Senecionine-d3 (IS-9) | 339.3 | 40 | 94 | 40 | 120 | 30 |  |  | 10.25 | 3 |  |
| Senecionine N-oxide | 352.2 | 40 | 94 | 40 | 120 | 30 | 136 | 30 | 6.70 ^c^ | 1 | IS-10 |
| Senecionine N-oxide-d3 (IS-10) | 355.2 | 40 | 94 | 40 | 120 | 30 |  |  | 6.70 | 1 |  |
| Seneciphylline | 334.2 | 40 | 120 | 30 | 138 | 30 | 94 | 40 | 9.25 | 3 | IS-9 |
| Seneciphylline N-oxide | 350.2 | 40 | 94 | 40 | 138 | 30 | 118 | 30 | 5.80 | 1 | IS-10 |
| Senecivernine | 336.2 | 40 | 94 | 40 | 120 | 30 | 138 | 30 | 10.40 | 3 | IS-9 |
| Senecivernine N-oxide | 352.2 | 40 | 94 | 40 | 120 | 30 | 136 | 30 | 6.75 ^c^ | 1 | IS-10 |
| Senkirkine | 366.2 | 30 | 122 | 30 | 168 | 25 |  |  | 7.00 | 1 | IS-4 |
| Spartioidine | 334.2 | 40 | 120 | 30 | 138 | 30 | 94 | 40 | 8.95 | 3 | IS-9 |
| Spartioidine N-oxide | 350.2 | 40 | 94 | 40 | 120 | 30 | 118 | 30 | 5.70 | 1 | IS-10 |
| Usaramine | 352.2 | 40 | 94 | 40 | 120 | 30 | 138 | 30 | 8.30 | 1 | IS-4 |
| Usaramine N-oxide | 368.2 | 40 | 94 | 40 | 120 | 30 | 119 | 30 | 5.25 | 2 | IS-5 |

a,b,c Isomeric compounds that are not (fully) chromatographically separated

**Table S5:** Preparation of feed materials: homogeneity test experiment with 10 selected PAs. N=10 for all materials.

|  | **Europine** | **Heliotrine** | **Seneci-phylline** | **Sene-cionine** | **Echimidine** | **Europine-N-oxide** | **Heliotrine-N-oxide** | **Seneci-phylline-N-oxide** | **Sene-cionine-N-oxide** | **Echimidine-N-oxide** |  | **Avg. PA FBs** | **Avg. PA NOs** | **Avg. PA Total** |
| --- | --- | --- | --- | --- | --- | --- | --- | --- | --- | --- | --- | --- | --- | --- |
| Conc. spiked (µg/kg) | 1000 | 1000 | 1000 | 1000 | 1000 | 1000 | 1000 | 1000 | 1000 | 1000 |  | 1000 | 1000 | 1000 |
| Conc. recovered (µg/kg) | 890 | 898 | 1104 | 991 | 856 | 993 | 854 | 1004 | 896 | 1041 |  | 948 | 958 | 953 |
| SD (µg/kg) | 31 | 37 | 58 | 66 | 95 | 31 | 43 | 34 | 26 | 54 |  | 26 | 23 | 19 |
| RSDr (%) | 3.4% | 4.2% | 5.3% | 6.7% | 11.1% | 3.1% | 5.0% | 3.4% | 2.9% | 5.2% |  | 2.7% | 2.4% | 2.0% |
|  |  |  |  |  |  |  |  |  |  |  |  |  |  |  |
| Recovery (%) | 89.0% | 89.8% | 110.4% | 99.1% | 85.6% | 99.3% | 85.4% | 100.4% | 89.6% | 104.1% |  | 94.8% | 95.8% | 95.3% |

**Table S6:** Measured levels of PA FBs and PA NOs (µg/kg DM ± SD) in starting feeds, diet residue and larvae. Percentage of PA FBs and PA NOs in the materials and the overall recovery percentage. N=3 for all materials, except for BSF diet residue and larvae phase 1 (N=2). The recovery is based on the levels measured in the starting feeds.

| **Material 1**  **(*J. vulgaris*)** | **Target level feed (µg/kg)** | **BSF feed (µg/kg)** | **BSF residue phase 1 (µg/kg)** | **BSF residue phase 2 (µg/kg)** | **BSF larvae phase 1 (µg/kg)** | **BSF larvae phase 2 (µg/kg)** |  | **LMW feed (µg/kg)** | **LMW residue (µg/kg)** | **LMW larvae (µg/kg)** |
| --- | --- | --- | --- | --- | --- | --- | --- | --- | --- | --- |
| Erucifoline | 550 | 627±17 | 378±55 | 218±49 | 35.2±8.5 | 15.1±1.4 |  | 580±36 | 590±29 | 8.7±0.7 |
| Integerrimine | 30 | 51.0±0.8 | 104±2.0 | 68.9±14.4 | 9.3±1.4 | 0±0 |  | 50.9±2.4 | 107±8.0 | 0±0 |
| Jacobine | 260 | 304±3.8 | 216±25 | 161±33 | 25.9±7.1 | 18.9±3.4 |  | 232±11 | 162±9.3 | 4.3±0.4 |
| Jacoline | 80 | 68.8±13.2 | 66.7±9.1 | 29.7±6.1 | 6.5±1.8 | 5.2±0.9 |  | 86.1±4.4 | 158±6.2 | 11.4±0.7 |
| Jaconine | 20 | 21.9±3.3 | 0±0 | 0±0 | 0±0 | 0±0 |  | 84.5±3.2 | 251±8.3 | 6.6±0.7 |
| Retrorsine | 40 | 48.7±1.7 | 98.3±8.3 | 58.3±19.6 | 6.1±2.8 | 2.7±0.7 |  | 46.6±4.1 | 93.5±7.1 | 0±0 |
| Riddelliine | 10 | 11.1±1.1 | 22.8±4.1 | 10.0±3.4 | 0±0 | 0±0 |  | 0±0 | 40.5±6.4 | 0±0 |
| Senecionine | 190 | 311±38 | 366±1.1 | 241±35 | 35.8±3.9 | 8.0±0.9 |  | 247±7.9 | 395±22 | 4.1±0.3 |
| Seneciphylline | 170 | 248±34 | 251±4.2 | 147±24 | 23.2±1.1 | 4.4±0.2 |  | 219±1.7 | 290±24 | 5.9±1.1 |
| Usaramine | 0 | 0±0 | 19.0±3.5 | 0±0 | 0±0 | 0±0 |  | 0±0 | 14.0±2.9 | 0±0 |
| Erucifoline N-oxide | 1160 | 1090±69 | 0±0 | 0±0 | 0±0 | 0±0 |  | 1102±26 | 369±45 | 0±0 |
| Integerrimine N-oxide | 240 | 189±4.2 | 0±0 | 0±0 | 0±0 | 0±0 |  | 243±4.0 | 80.9±5.5 | 0±0 |
| Jacobine N-oxide | 870 | 763±54 | 0±0 | 0±0 | 0±0 | 0±0 |  | 597±6.2 | 109±12 | 0±0 |
| Jacoline N-oxide | 120 | 121±49 | 0±0 | 0±0 | 0±0 | 0±0 |  | 138±8.0 | 66.9±12.1 | 0±0 |
| Jaconine N-oxide | 0 | 17.3±1.3 | 0±0 | 0±0 | 0±0 | 0±0 |  | 200±8.5 | 131±16 | 0±0 |
| Retrorsine N-oxide | 170 | 152±6.3 | 0±0 | 0±0 | 0±0 | 0±0 |  | 167±2.7 | 61.3±5.6 | 0±0 |
| Riddelliine N-oxide | 40 | 38.7±3.2 | 0±0 | 0±0 | 0±0 | 0±0 |  | 40.4±0.9 | 27.3±3.4 | 0±0 |
| Senecionine N-oxide | 840 | 659±22 | 0±0 | 0±0 | 0±0 | 0±0 |  | 818±14 | 229±24 | 0±0 |
| Seneciphylline N-oxide | 310(620)^a^ | 309±12 | 0±0 | 0±0 | 0±0 | 0±0 |  | 352±4.7 | 203±19 | 0±0 |
| Usaramine N-oxide | 30 | 25.5±2 | 0±0 | 0±0 | 0±0 | 0±0 |  | 28.8±0.6 | 11.9±1.4 | 0±0 |
| Sum PA FBs | 1350 | 1691±104 | 1525±108 | 934±166 | 142±24 | 54.4±5.1 |  | 1546±62 | 2100±116 | 41.1±3 |
| Sum PA NOs | 3780 | 3364±213 | 0±0 | 0±0 | 0±0 | 0±0 |  | 3686±67 | 1289±137 | 0±0 |
| Sum Total PAs | 5130 | 5055±314 | 1525±108 | 934±166 | 142±24 | 54.4±5.1 |  | 5232±86 | 3389±170 | 41.1±3 |
| % PA FBs | 26.3% | 33.5% | 100% | 100% | 100% | 100% |  | 29.5% | 62.0% | 100% |
| % PA NOs | 73.7% | 66.5% | 0% | 0% | 0% | 0% |  | 71.5% | 38.0% | 0% |
| % Recovery PA FBs |  |  | 90.2% | 55.2% | 8.4% | 3.2% |  |  | 135.8% | 2.7% |
| % Recovery PA NOs |  |  | 0% | 0% | 0% | 0% |  |  | 35.0% | 0% |
| % Recovery Total PAs |  |  | 30.2% | 18.5% | 2.8% | 1.1% |  |  | 64.8% | 0.8% |

a Due to an error made in the preparation of the stock solution, the concentration in the mixed spiking solution was a factor 2 lower.

| **Material 2**  **(*S. vulgaris*)** | **Target level feed (µg/kg)** | **BSF feed (µg/kg)** | **BSF residue phase 1 (µg/kg)** | **BSF residue phase 2 (µg/kg)** | **BSF larvae phase 1 (µg/kg)** | **BSF larvae phase 2 (µg/kg)** |  | **LMW feed (µg/kg)** | **LMW residue (µg/kg)** | **LMW larvae (µg/kg)** |
| --- | --- | --- | --- | --- | --- | --- | --- | --- | --- | --- |
| Integerrimine | 50 | 97.4±1.3 | 594±4.3 | 216±11 | 48.9±4 | 2.9±0.9 |  | 75.4±1.9 | 294±12 | 0±0 |
| Retrorsine | 130 | 198±1.3 | 1852±266 | 436±106 | 176±4.7 | 16.1±2.0 |  | 187±1.4 | 676±60 | 4.6±0.2 |
| Riddelliine | 30 | 40.1±3.6 | 355±0.9 | 73.5±9.7 | 25.8±0.5 | 3±1.2 |  | 42.7±0.6 | 254±22 | 1.8±1.6 |
| Senecionine | 170 | 378±13 | 1243±35 | 379±64 | 114±5.9 | 10.1±2.0 |  | 219±7.9 | 671±31 | 4±1 |
| Seneciphylline | 620 | 724±43 | 3398±34 | 1004±106 | 305±3.2 | 19.4±0.9 |  | 911±42 | 1624±225 | 20.4±2.5 |
| Senecivernine | 20 | 46.6±2.0 | 144±5.4 | 42.5±0.5 | 11.2±4.8 | 0±0 |  | 34.4±3.1 | 86.6±4.2 | 0±0 |
| Spartioidine | 90 | 95.2±2.5 | 103±1.7 | 41.4±2.3 | 9.5±0.3 | 0±0 |  | 45.0±1.9 | 34.8±2.0 | 0±0 |
| Usaramine | 0 | 0±0 | 58.2±2.0 | 5.8±5.0 | 4.4±0.5 | 0±0 |  | 0±0 | 18.4±1.0 | 0±0 |
| Integerrimine N-oxide | 710 | 606±16.5 | 3.3±0.4 | 0±0 | 0±0 | 0±0 |  | 818±28 | 252±20 | 0±0 |
| Retrorsine N-oxide | 1720 | 1729±57 | 6.2±0.0 | 4.4±4.0 | 0±0 | 0±0 |  | 1993±76 | 660±62 | 0±0 |
| Riddelliine N-oxide | 270 | 257±8.7 | 0±0 | 0±0 | 0±0 | 0±0 |  | 309±8.2 | 252±27 | 0±0 |
| Senecionine N-oxide | 1660 | 1308±19 | 5.0±0.1 | 5.3±1.5 | 0±0 | 0±0 |  | 1504±87 | 457±27 | 2.2±1.9 |
| Seneciphylline N-oxide | 2450(4900)^a^ | 2366±122 | 7.0±0.3 | 10.2±4.2 | 0±0 | 0±0 |  | 2734±70 | 1455±125 | 0±0 |
| Senecivernine N-oxide | 150 | 124±12 | 0±0 | 0±0 | 0±0 | 0±0 |  | 238±20 | 70.4±3.0 | 0±0 |
| Spartioidine N-oxide | 0 | 0±0 | 0±0 | 0±0 | 0±0 | 0±0 |  | 0±0 | 0±0 | 0±0 |
| Usaramine N-oxide | 20 | 32.2±2.0 | 0±0 | 0±0 | 0±0 | 0±0 |  | 41.7±0.9 | 15.9±1.3 | 0±0 |
| Sum PA FBs | 1110 | 1579±49 | 7748±341 | 2198±207 | 695±2.5 | 51.5±1.1 |  | 1515±38 | 3659±299 | 30.8±4.5 |
| Sum PA NOs | 6980 | 6422±204 | 21.6±0.8 | 19.9±9.5 | 0±0 | 0±0 |  | 7638±226 | 3162±258 | 2.2±1.9 |
| Sum Total PAs | 8090 | 8001±159 | 7769±340 | 2218±205 | 695±2.5 | 51.5±1.1 |  | 9154±260 | 6821±556 | 33.0±6.0 |
| % PA FBs | 13.7% | 19.7% | 99.7% | 99.1% | 100% | 100% |  | 16.6% | 53.6% | 93.3% |
| % PA NOs | 86.3% | 80.3% | 0.3% | 0.9% | 0% | 0% |  | 83.4% | 46.4% | 6.7% |
| % Recovery PA FBs |  |  | 490% | 139.1% | 44.0% | 3.3% |  |  | 241% | 2.0% |
| % Recovery PA NOs |  |  | 0.3% | 0.3% | 0% | 0% |  |  | 41.4% | 0% |
| % Recovery Total PAs |  |  | 97.1% | 27.7% | 8.7% | 0.6% |  |  | 74.5% | 0.4% |

*a Due to an error made in the preparation of the stock solution, the concentration in the mixed spiking solution was a factor 2 lower.*

| **Material 3**  **(*S. inaequidens*)** | **Target level feed (µg/kg)** | **BSF feed (µg/kg)** | **BSF residue phase 1 (µg/kg)** | **BSF residue phase 2 (µg/kg)** | **BSF larvae phase 1 (µg/kg)** | **BSF larvae phase 2 (µg/kg)** |  | **LMW feed (µg/kg)** | **LMW residue (µg/kg)** | **LMW larvae (µg/kg)** |
| --- | --- | --- | --- | --- | --- | --- | --- | --- | --- | --- |
| Integerrimine | 180 | 606±17 | 2807±254 | 2312±674 | 435±50 | 19.6±1.8 |  | 471±9.9 | 1300±104 | 11.8±1.4 |
| Retrorsine | 2500 | 2838±198 | 17770±27 | 12492±3205 | 2693±151 | 262±70 |  | 2384±97 | 7539±927 | 62.3±8.5 |
| Riddelliine | 0 | 0±0 | 11.1±0.1 | 5.9±2.8 | 0±0 | 0±0 |  | 0±0 | 7.8±1.2 | 0±0 |
| Sceleratine | 130 | 131±7.6 | 195±15 | 65.3±28.9 | 40.0±0.4 | 3.6±0.2 |  | 157±6.2 | 235±35 | 7.7±1.1 |
| Senecionine | 310 | 371±15 | 1028±89 | 640±197 | 155±6.3 | 10.9±0.7 |  | 247±3.0 | 658±34 | 6.5±0.6 |
| Senecivernine | 870 | 1832±73 | 6334±514 | 3711±1088 | 777±57 | 57.6±8.6 |  | 1172±16 | 4105±336 | 24.2±4.6 |
| Senkirkine | 6650 | 5022±356 | 6135±171 | 3090±308 | 783±16 | 29.9±2.8 |  | 5619±298 | 6629±787 | 25.0±3.6 |
| Usaramine | 330 | 528±15 | 3915±88 | 2821±862 | 577±19 | 81.4±14.6 |  | 541±12 | 1521±167 | 16.1±0.6 |
| Integerrimine N-oxide | 1670 | 4817±115 | 16.8±6.7 | 22.5±5.7 | 0±0 | 0±0 |  | 4525±340 | 2043±4 | 9.1±1.4 |
| Retrorsine N-oxide | 23800 | 22876±79 | 61.8±26.1 | 94.7±25.5 | 5.8±0.1 | 0±0 |  | 21238±1828 | 11230±989 | 47.3±9.0 |
| Riddelliine N-oxide | 0 | 11.9±0.4 | 0±0 | 0±0 | 0±0 | 0±0 |  | 13.8±0.7 | 7.1±0.4 | 0±0 |
| Sceleratine N-oxide | 340 | 328±9.4 | 0±0 | 0±0 | 0±0 | 0±0 |  | 365±26 | 178±9.0 | 0±0 |
| Senecionine N-oxide | 5000 | 1237±83 | 2.6±3.7 | 4.5±0.8 | 0±0 | 0±0 |  | 1352±130 | 498±30.1 | 4.4±1.1 |
| Senecivernine N-oxide | 8700 | 8849±99 | 21.6±7.5 | 31.9±9.2 | 0±0 | 0±0 |  | 7778±585 | 3072±233 | 11.4±1.8 |
| Usaramine N-oxide | 4100 | 3502±32 | 9.8±3.2 | 12.8±4.3 | 0±0 | 0±0 |  | 4083±284 | 2225±182 | 8.3±1.8 |
| Sum PA FBs | 10970 | 11328±666 | 38200±1158 | 25138±6239 | 5460±73 | 465±89 |  | 10592±391 | 21994±2356 | 154±20 |
| Sum PA NOs | 43610 | 41620±202 | 113±47 | 166±45 | 5.8±0.1 | 0±0 |  | 39354±3120 | 19252±1306 | 80.5±14.8 |
| Sum Total PAs | 54580 | 52948±571 | 38312±1206 | 25304±6199 | 5466±73 | 465±89 |  | 49946±3504 | 41247±3658 | 234±33 |
| % PA FBs | 20.1% | 21.4% | 99.7% | 99.3% | 99.7% | 100% |  | 21.2% | 53.3% | 65.8% |
| % PA NOs | 79.9% | 78.6% | 0.3% | 0.7% | 0.3% | 0% |  | 78.8% | 46.7% | 34.2% |
| % Recovery PA FBs |  |  | 337% | 222% | 48.2% | 4.1% |  |  | 208% | 1.4% |
| % Recovery PA NOs |  |  | 0.3% | 0.4% | 0% | 0% |  |  | 48.9% | 0.2% |
| % Recovery Total PAs |  |  | 72.4% | 47.8% | 10.3% | 0.9% |  |  | 82.6% | 0.5% |

| **Material 4**  **(*E. vulgare*)** | **Target level feed (µg/kg)** | **BSF feed (µg/kg)** | **BSF residue phase 1 (µg/kg)** | **BSF residue phase 2 (µg/kg)** | **BSF larvae phase 1 (µg/kg)** | **BSF larvae phase 2 (µg/kg)** |  | **LMW feed (µg/kg)** | **LMW residue (µg/kg)** | **LMW larvae (µg/kg)** |
| --- | --- | --- | --- | --- | --- | --- | --- | --- | --- | --- |
| Echimidine | 370 | 680±19 | 2007±250 | 1035±354 | 329±48 | 24.6±5.0 |  | 546±16 | 2350±242 | 9.9±2.7 |
| Europine | 0 | 0±0 | 5.8±0.1* | 0±0 | 5.6±0.4* | 0±0 |  | 0±0 | 0±0 | 0±0 |
| Heliotrine | 0 | 0±0 | 7.0±0.5* | 0±0 | 0±0 | 0±0 |  | 0±0 | 0±0 | 0±0 |
| Lasiocarpine | 0 | 0±0 | 3.5±0.7* | 0±0 | 0±0 | 0±0 |  | 0±0 | 0±0 | 0±0 |
| Echimidine N-oxide | 5100 | 4464±192 | 6.8±1.2 | 4.4±2.1 | 14.8±1.7 | 0±0 |  | 4331±614 | 1598±101 | 0±0 |
| Sum PA FBs | 370 | 680±19 | 2023±250 | 1035±354 | 334±48 | 24.6±5.0 |  | 546±16 | 2350±242 | 9.9±2.7 |
| Sum PA NOs | 5100 | 4464±192 | 6.8±1.2 | 4.4±2.1 | 14.8±1.7 | 0±0 |  | 4331±614 | 1599±100 | 0±0 |
| Sum Total PAs | 5470 | 5144±177 | 2030±249 | 1039±356 | 349±46 | 24.6±5.0 |  | 4876±603 | 3949±162 | 9.9±2.7 |
| % PA FBs | 6.8% | 13.2% | 99.7% | 99.6% | 95.7% | 100.0% |  | 11.2% | 59.5% | 100.0% |
| % PA NOs | 93.2% | 86.8% | 0.3% | 0.4% | 4.2% | 0.0% |  | 88.8% | 40.5% | 0.0% |
| % Recovery PA FBs |  |  | 297% | 222% | 49.1% | 3.6% |  |  | 431% | 1.8% |
| % Recovery PA NOs |  |  | 0.2% | 0.4% | 0.3% | 0% |  |  | 36.9% | 0% |
| % Recovery Total PAs |  |  | 39.5% | 47.8% | 6.8% | 0.5% |  |  | 81.0% | 0.2% |

* Low level contamination (from material 5).

| **Material 5**  **(*H. europaeum*)** | **Target level feed (µg/kg)** | **BSF feed (µg/kg)** | **BSF residue phase 1 (µg/kg)** | **BSF residue phase 2 (µg/kg)** | **BSF larvae phase 1 (µg/kg)** | **BSF larvae phase 2 (µg/kg)** |  | **LMW feed (µg/kg)** | **LMW residue (µg/kg)** | **LMW larvae (µg/kg)** |
| --- | --- | --- | --- | --- | --- | --- | --- | --- | --- | --- |
| Echinatine | 0 | 0±0 | 157±7.2 | 225±17 | 25.2±1.8 | 25.7±2.2 |  | 1.8±0.1 | 38.9±5.3 | 7.9±0.7 |
| Europine | 230 | 288±23 | 5153±7 | 5027±406 | 846±9.3 | 798±106 |  | 293±4.5 | 1503±87 | 679±52 |
| Heliosupine | 30 | 45.0±13.7 | 113±3.2 | 53.7±6.0 | 7.6±1.2 | 0±0 |  | 29.8±3.4 | 80.7±5.8 | 0±0 |
| Heliotrine | 420 | 582±38 | 3711±59 | 2438±376 | 550±29 | 206±17 |  | 533±13 | 2250±68 | 94.3±18.5 |
| Lasiocarpine | 270 | 504±40 | 2349±138 | 1043±105 | 180±4.8 | 20.2±1.6 |  | 411±9.9 | 1809±88 | 16.1±2.4 |
| Rinderine | 0 | 3.7±0.2 | 268±8.2 | 364±27 | 49.9±1.9 | 52.9±5.5 |  | 3.5±0.2 | 154±13 | 34.8±5.9 |
| Echinatine N-oxide | 130 | 142±11 | 0±0 | 0±0 | 0±0 | 0±0 |  | 137±2.8 | 95.5±6.3 | 0±0 |
| Europine N-oxide | 5800 | 6397±327 | 8.7±0.7 | 12.4±3.7 | 46.3±33.3 | 0±0 |  | 6210±402 | 4253±339 | 31.6±4.9 |
| Heliosupine N-oxide | 170 | 173±15 | 0±0 | 0±0 | 0±0 | 0±0 |  | 180±7.4 | 117±6.2 | 0±0 |
| Heliotrine N-oxide | 8000 | 9376±498 | 8.8±0.5 | 7.6±2.9 | 55.3±40.8 | 0±0 |  | 7792±526 | 5939±397 | 26.0±2.3 |
| Lasiocarpine N-oxide | 4300 | 3729±321 | 7.3±0 | 5.5±1.7 | 19.5±12.2 | 0±0 |  | 3490±165 | 1247±42 | 7.1±1 |
| Rinderine N-oxide | 320 | 343±31 | 0±0 | 0±0 | 0±0 | 0±0 |  | 347±5.2 | 567±18 | 0±0 |
| Sum PA FBs | 950 | 1422±81 | 11751±104 | 9151±920 | 1659±48 | 1104±129 |  | 1272±23 | 5837±241 | 832±69 |
| Sum PA NOs | 18720 | 20159±520 | 24.8±1.3 | 25.5±8.2 | 121±86 | 0±0 |  | 18157±1062 | 12218±770 | 64.6±7.9 |
| Sum Total PAs | 19670 | 21581±445 | 11776±103 | 9177±922 | 1780±134 | 1104±129 |  | 19429±1085 | 18054±1011 | 896±66 |
| % PA FBs | 4.8% | 6.6% | 99.8% | 99.7% | 93.2% | 100.0% |  | 6.5% | 32.3% | 92.9% |
| % PA NOs | 95.2% | 93.4% | 0.2% | 0.3% | 6.8% | 0.0% |  | 93.5% | 67.7% | 7.2% |
| % Recovery PA FBs |  |  | 826% | 643% | 116.7% | 77.6% |  |  | 459% | 65.4% |
| % Recovery PA NOs |  |  | 0.1% | 0.1% | 0.6% | 0% |  |  | 67.3% | 0.4% |
| % Recovery Total PAs |  |  | 54.6% | 42.5% | 8.2% | 5.1% |  |  | 92.9% | 4.6% |

| **Material 6**  **(Mix of 5 herbs)** | **Target level feed (µg/kg)** | **BSF feed (µg/kg)** | **BSF residue phase 1 (µg/kg)** | **BSF residue phase 2 (µg/kg)** | **BSF larvae phase 1 (µg/kg)** | **BSF larvae phase 2 (µg/kg)** |  | **LMW feed (µg/kg)** | **LMW residue (µg/kg)** | **LMW larvae (µg/kg)** |
| --- | --- | --- | --- | --- | --- | --- | --- | --- | --- | --- |
| Echimidine | 74 | 146±5.8 | 459±42 | 282±93 | 35.9±1.3 | 6.3±1.3 |  | 118±6.7 | 333±59 | 2.8±0.6 |
| Echinatine | 0 | 0±0 | 27.8±1.8 | 45.7±9.8 | 5.2±0.5 | 6.8±2.3 |  | 0±0 | 10.9±2.8 | 0±0 |
| Erucifoline | 110 | 122±6.1 | 85.4±0.5 | 41.2±19 | 8.7±0.6 | 0±0 |  | 123±10 | 127±10 | 0±0 |
| Europine | 46 | 70.8±0.1 | 1013±48 | 1204±100 | 199±11 | 168±21 |  | 68.7±4.8 | 313±47 | 163±11 |
| Heliosupine | 6 | 10.3±0.6 | 31.2±3.1 | 10.6±2.1 | 0±0 | 0±0 |  | 6.6±1 | 28.0±7.4 | 0±0 |
| Heliotrine | 84 | 143±0.8 | 889±52 | 576±129 | 103±6.3 | 44.7±8.6 |  | 128±5.9 | 460±79 | 21.9±3.2 |
| Integerrimine | 78 | 197±7.1 | 530±38 | 405±169 | 50.3±5.6 | 4.2±0.1 |  | 149±10 | 435±54 | 2.8±0.6 |
| Jacobine | 52 | 60.5±2.3 | 50.8±0.3 | 26.2±10.6 | 7.2±0.3 | 0±0 |  | 51.9±1.9 | 34.1±4.3 | 0±0 |
| Jacoline | 16 | 17.0±1.8 | 16.0±1.1 | 5.3±1.5 | 0±0 | 0±0 |  | 17.2±0.9 | 29.7±4.2 | 3.6±0.1 |
| Jaconine | 4 | 5.0±1.1 | 0±0 | 0±0 | 0±0 | 0±0 |  | 14.3±3.3 | 49.6±7.7 | 0±0 |
| Lasiocarpine | 54 | 104±0.7 | 552±16 | 188±45 | 36.5±0.0 | 3.7±0.1 |  | 83.6±2.0 | 317±58 | 0±0 |
| Retrorsine | 534 | 837±29 | 2874±106 | 1974±590 | 218±15 | 59.8±6.6 |  | 766±32 | 1757±193 | 16.8±2 |
| Riddelliine | 6 | 10.1±0.6 | 33.3±1.5 | 22.2±7.8 | 0±0 | 0±0 |  | 10.1±1.6 | 63.9±11.5 | 0±0 |
| Rinderine | 0 | 0±0 | 51.9±0.4 | 82.1±15.1 | 10.7±0.1 | 10.4±0.8 |  | 0±0 | 43.9±9.0 | 10.6±0.9 |
| Sceleratine | 26 | 27.3±1.8 | 18.2±0.8 | 4.3±2.0 | 0±0 | 0±0 |  | 34±5.2 | 47.1±6.8 | 2.6±0.2 |
| Senecionine | 108 | 247±0.8 | 379±27 | 246±122 | 32.5±3.8 | 5.1±0.8 |  | 167±5.1 | 348±47 | 4.3±0.8 |
| Seneciphylline | 158 | 302±11 | 435±19 | 249±112 | 40.9±1.7 | 4.2±0.5 |  | 238±31 | 403±41 | 9.1±1.2 |
| Senecivernine | 178 | 532±18 | 972±31 | 543±237 | 69.2±5.1 | 10.3±0.8 |  | 386±11 | 739±104 | 5.0±0.4 |
| Senkirkine | 1330 | 1013±20 | 779±25 | 259±27 | 58.8±0.9 | 3.4±0.5 |  | 1015±52 | 1042±119 | 0±0 |
| Spartioidine | 18 | 19.0±1.0 | 10.4±0.8 | 9.2±3.1 | 0±0 | 0±0 |  | 20.4±2.9 | 12.2±0.7 | 0±0 |
| Usaramine | 66 | 131±4.3 | 516±12 | 324±88 | 44.3±4.8 | 15.8±1.9 |  | 111±3.5 | 376±62 | 5.4±0.6 |
| Echimidine N-oxide | 1020 | 898±59 | 3.9±0.5 | 0±0 | 0±0 | 0±0 |  | 869±59 | 358±27 | 0±0 |
| Echinatine N-oxide | 26 | 28.3±1.4 | 0±0 | 0±0 | 0±0 | 0±0 |  | 27.8±1.7 | 17.9±2.6 | 0±0 |
| Erucifoline N-oxide | 232 | 215±0.6 | 0±0 | 0±0 | 0±0 | 0±0 |  | 235±1.7 | 76.8±8.4 | 0±0 |
| Europine N-oxide | 1160 | 1277±36 | 0±0 | 0±0 | 0±0 | 0±0 |  | 1389±27 | 774±75 | 3.8±0.8 |
| Heliosupine N-oxide | 34 | 38.0±1.1 | 0±0 | 0±0 | 0±0 | 0±0 |  | 37.4±2.4 | 23.4±1.0 | 0±0 |
| Heliotrine N-oxide | 1600 | 2016±16 | 0±0 | 0±0 | 0±0 | 0±0 |  | 1755±63 | 1237±170 | 0±0 |
| Integerrimine N-oxide | 1190 | 865±23 | 11.7±0.1 | 5.7±2.2 | 0±0 | 0±0 |  | 1217±36 | 396±56 | 0±0 |
| Jacobine N-oxide | 174 | 137±3.8 | 0±0 | 0±0 | 0±0 | 0±0 |  | 143±2.0 | 24.1±2.4 | 0±0 |
| Jacoline N-oxide | 24 | 36.6±1.2 | 0±0 | 0±0 | 0±0 | 0±0 |  | 27.6±1.8 | 14.5±2.2 | 0±0 |
| Jaconine N-oxide | 0 | 5.2±0.8 | 0±0 | 0±0 | 0±0 | 0±0 |  | 34.9±1.8 | 26.5±2.4 | 0±0 |
| Lasiocarpine N-oxide | 860 | 892±33 | 3.1±0.3 | 0±0 | 0±0 | 0±0 |  | 870±18 | 316±29 | 0±0 |
| Retrorsine N-oxide | 5138 | 5308±190 | 26.6±1.6 | 18.2±5.2 | 0±0 | 0±0 |  | 5730±342 | 2026±290 | 3.3±1.1 |
| Riddelliine N-oxide | 62 | 62.5±2.3 | 0±0 | 0±0 | 0±0 | 0±0 |  | 75.6±1 | 54.1±5.3 | 0±0 |
| Rinderine N-oxide | 64 | 68.1±2.1 | 0±0 | 0±0 | 0±0 | 0±0 |  | 71.0±2.5 | 112±6.8 | 0±0 |
| Sceleratine N-oxide | 68 | 62.4±1.6 | 0±0 | 0±0 | 0±0 | 0±0 |  | 71.3±2.2 | 28.7±4.3 | 0±0 |
| Senecionine N-oxide | 834 | 542±20 | 4.9±0.5 | 0±0 | 0±0 | 0±0 |  | 802±17 | 205±18 | 0±0 |
| Seneciphylline N-oxide | 552 | 493±19 | 3.2±0.1 | 0±0 | 0±0 | 0±0 |  | 604±3.4 | 345±44 | 0±0 |
| Senecivernine N-oxide | 1770 | 1430±51 | 13.9±0.3 | 5.8±3.4 | 0±0 | 0±0 |  | 1758±142 | 526±59 | 0±0 |
| Spartioidine N-oxide | 0 | 0±0 | 0±0 | 0±0 | 0±0 | 0±0 |  | 0±0 | 0±0 | 0±0 |
| Usaramine N-oxide | 830 | 797±22 | 3.2±0.4 | 0±0 | 0±0 | 0±0 |  | 952±15 | 368±48 | 0±0 |
| Sum PA FBs | 2948 | 3993±48 | 9722±214 | 6496±1681 | 920±47 | 343±39 |  | 3508±132 | 6969±901 | 248±19 |
| Sum PA NOs | 15638 | 15172±358 | 70.5±1.0 | 29.7±10.7 | 0±0 | 0±0 |  | 16670±594 | 6927±825 | 7.1±1.8 |
| Sum Total PAs | 18586 | 19165±345 | 9792±215 | 6526±1691 | 920±47 | 343±39 |  | 20177±490 | 13896±1725 | 255±18 |
| % PA FBs | 15.9% | 20.8% | 99.3% | 99.5% | 100.0% | 100.0% |  | 17.4% | 50.2% | 97.3% |
| % PA NOs | 84.1% | 79.2% | 0.7% | 0.5% | 0.0% | 0.0% |  | 82.6% | 49.8% | 2.8% |
| % Recovery PA FBs |  |  | 244% | 162.7% | 162.7% | 8.6% |  |  | 198.7% | 7.1% |
| % Recovery PA NOs |  |  | 0.5% | 0.2% | 0.2% | 0% |  |  | 41.6% | 05 |
| % Recovery Total PAs |  |  | 51.1% | 34.0% | 34.0% | 1.8% |  |  | 68.9% | 1.3% |

| **Material 7 & 8**  **(Atropine & scopolamine)** | **Target level feed (µg/kg)** | **BSF feed (µg/kg)** | **BSF residue phase 1 (µg/kg)** | **BSF residue phase 2 (µg/kg)** | **BSF larvae phase 1 (µg/kg)** | **BSF larvae phase 2 (µg/kg)** |  | **LMW feed (µg/kg)** | **LMW residue (µg/kg)** | **LMW larvae (µg/kg)** |
| --- | --- | --- | --- | --- | --- | --- | --- | --- | --- | --- |
| Atropine | 4000 | 3537±60 | 2256±99 | 1377±312 | 212±7.0 | 30.6±2 |  | 3361±186 | 1921±110 | 14.0±2.0 |
| Scopolamine | 1000 | 1143±40 | 1584±153 | 2038±374 | 113±0.5 | 32.1±1.6 |  | 940±20 | 946±31 | 6.4±1.2 |
| % Recovery atropine |  |  | 63.9% | 38.9% | 6.1% | 0.9% |  |  | 57.2% | 0.4% |
| % Recovery scopolamine |  |  | 138.5% | 178.3% | 9.9% | 2.8% |  |  | 100.6% | 0.7% |

**Table S7:** Stability experiment of BSF and LMW feed materials, simulating the conditions used during insect rearing. BSF feeds were mixed with two volumes of water and were incubated for 7 days at a temperature of 28°C. LMW feeds were mixed with one volume of water and incubated at 28°C. Incremental portions were added each day for a total of 21 days. Concentrations in µg/kg DM (N=1).

| **Material** |  | **1**  **(*J. vulgaris*)** | **2**  **(*S. vulgaris*)** | **3**  **(*S. inaequidens*)** | **4**  **(*E. vulgare*)** | **5**  **(*H. europaeum*)** | **6**  **(5 Herbs mix)** | **7**  **(Atropine)** | **8 (Scopolamine)** |
| --- | --- | --- | --- | --- | --- | --- | --- | --- | --- |
| BSF feed, | PA FBs (µg/kg) | 1661 | 1667 | 12422 | 1159 | 3513 | 4574 | - | - |
| T = 0 days | PA NOs (µg/kg) | 3140 | 6707 | 39705 | 3715 | 15259 | 11613 | - | - |
|  | Total (µg/kg) | 4800 | 8374 | 52127 | 4874 | 18772 | 16187 | 3067 | 1077 |
|  | % PA FBs | 34.6% | 19.9% | 23.8% | 23.8% | 18.7% | 28.3% | - | - |
|  | % PA NOs | 65.4% | 80.1% | 76.2% | 76.2% | 81.3% | 71.7% | - | - |
|  |  |  |  |  |  |  |  |  |  |
| BSF feed, | PA FBs (µg/kg) | 2688 | 4648 | 29138 | 5527 | 21978 | 10878 | - | - |
| T = 7 days ^a^ | PA NOs (µg/kg) | 45 | 438 | 1368 | 129 | 146 | 230 | - | - |
|  | Total (µg/kg) | 2733 | 5086 | 30506 | 5656 | 22124 | 11108 | 4188 | 1061 |
|  | % PA FBs | 98.4% | 91.4% | 95.5% | 97.7% | 99.3% | 97.9% | - | - |
|  | % PA NOs | 1.6% | 8.6% | 4.5% | 2.3% | 0.7% | 2.1% | - | - |
|  | % Recovery FBs | 161.9% | 279% | 235% | 477% | 626% | 238% | - | - |
|  | % Recovery NOs | 1.4% | 6.5% | 3.4% | 3.5% | 1.0% | 2.0% | - | - |
|  | % Recovery Total | 56.9% | 60.7% | 58.5% | 116.0% | 117.9% | 68.6% | 136.5% | 98.5% |
|  |  |  |  |  |  |  |  |  |  |
| BSF feed, | PA FBs (µg/kg) | 3268 | 5117 | 31491 | 5299 | 18289 | 11339 | - | - |
| T = 7 days ^b^ | PA NOs (µg/kg) | 28 | 24 | 41 | 36 | 36 | 33 | - | - |
|  | Total (µg/kg) | 3295 | 5141 | 31532 | 5335 | 18325 | 11372 | 4122 | 1043 |
|  | % PA FBs | 99.2% | 99.5% | 99.9% | 99.3% | 99.8% | 99.7% | - | - |
|  | % PA NOs | 0.8% | 0.5% | 0.1% | 0.7% | 0.2% | 0.3% | - | - |
|  | % Recovery FBs | 196.8% | 307% | 254% | 457% | 521% | 248% | - | - |
|  | % Recovery NOs | 0.9% | 0.4% | 0.1% | 1.0% | 0.2% | 0.3% | - | - |
|  | % Recovery Total | 68.6% | 61.4% | 60.5% | 109.5% | 97.6% | 70.3% | 134.4% | 96.8% |
|  |  |  |  |  |  |  |  |  |  |
| LMW feed, | PA FBs (µg/kg) | 1756 | 1951 | 17511 | 951 | 2956 | 4308 | - | - |
| T = 0 days | PA NOs (µg/kg) | 2606 | 5715 | 30854 | 3359 | 16091 | 11861 | - | - |
|  | Total (µg/kg) | 4361 | 7666 | 48365 | 4310 | 19047 | 16169 | 3472 | 841 |
|  | % PA FBs | 40.3% | 25.5% | 36.2% | 22.1% | 15.5% | 26.6% | - | - |
|  | % PA NOs | 59.7% | 74.5% | 63.8% | 77.9% | 84.5% | 73.4% | - | - |
|  |  |  |  |  |  |  |  |  |  |
| LMW feed, | PA FBs (µg/kg) | 1541 | 2879 | 16621 | 2524 | 6672 | 6220 | - | - |
| T = 21 days ^a^ | PA NOs (µg/kg) | 1223 | 2883 | 17395 | 1724 | 10291 | 6801 | - | - |
| (N=2) | Total (µg/kg) | 2764 | 5763 | 34017 | 4248 | 16963 | 13021 | 3126 | 775 |
|  | % PA FBs | 55.2% | 49.9% | 48.9% | 59.4% | 39.3% | 47.8% | - | - |
|  | % PA NOs | 44.8% | 50.1% | 51.1% | 40.6% | 60.7% | 52.2% | - | - |
|  | % Recovery FBs | 87.8% | 147.6% | 94.9% | 265% | 226% | 144.4% | - | - |
|  | % Recovery NOs | 46.9% | 50.5% | 56.4% | 51.3% | 64.0% | 57.3% | - | - |
|  | % Recovery Total | 63.4% | 75.2% | 70.3% | 98.6% | 89.1% | 80.5% | 90.0% | 92.2% |

a Lid not fully closed. b Lid closed.

**Table S8**: Yield, mean individual larval fresh weight (FW), and survival for the experiments with both phases of black soldier fly larvae (BSFL, phase 1 and phase 2) and lesser mealworm larvae (LMWL). Mean and standard deviation. Yield expressed as the total biomass of larvae after rinsing and drying. For the LMWL and BSFL-phase 1, mean individual larval weight was calculated as the mean number of counted larvae in up to N=3 samples for a given sample weight (~3 g for LMWL; ~1 g for BSFL-phase 1). For LMWL and BSFL-phase 1, survival rate is expressed as the extrapolated number of larvae for the yield (based on mean individual larval weight), as a percentage of the estimated number of larvae at the start of the experiment. For BSFL-phase 2, larval weight was calculated as the measured yield divided by the actual counted number of larvae at the end of the experiment. Survival was expressed as the counter number of larvae at the end of experiment as a percentage of the N=100 larvae with which the experiment was started.

|  | Black soldier fly larvae – Phase 1 | | | Black soldier fly larvae – Phase 2 | | | Lesser mealworm larvae | | |
| --- | --- | --- | --- | --- | --- | --- | --- | --- | --- |
| Treatment | **Yield**  **(g FW)** | **Larval weight (mg FW)** | **Survival**  **(%)** | **Yield**  **(g FW)** | **Larval weight (mg FW)** | **Survival**  **(%)** | **Yield**  **(g FW)** | **Larval weight (mg FW)** | **Survival**  **(%)** |
| 1 (*J. vulgaris*) | 211.5±13.1 | 15.9±0.9 | 49.0±4.8 | 13.4±0.2 | 134.6±2.4 | 99.7±0.6 | 91.7±2.5 | 17.7±0.5 | 88.3±3.5 |
| 2 (*S. vulgaris*) | 214.1±20.1 | 15.8±0.9 | 49.8±4.1 | 13.0±0.7 | 130.4±5.8 | 99.7±0.6 | 92.3±0.6 | 17.4±1.1 | 90.4±4.9 |
| 3 (*S. inaequidens*) | 195.6±31.8 | 13.7±0.8 | 52.6±6.9 | 12.5±0.3 | 125.3±3.2 | 99.7±0.6 | 86.0±2.0 | 16.2±0.3 | 90.5±0.7 |
| 4 (*E. vulgare*) | 197.4±4.7 | 18.2±3.1 | 40.2±5.7 | 13.1±0.1 | 131.9±1.5 | 99.7±0.6 | 87.7±3.5 | 16.4±0.7 | 91.1±2.6 |
| 5 (*H. europaeum*) | 225.1±7.9 | 15.9±1.4 | 52.1±4.8 | 13.3±0.3 | 133.6±4.0 | 99.7±0.6 | 92.0±6.1 | 18.1±0.9 | 86.2±1.4 |
| 6 (5 Herbs mix) | 219.9±6.9 | 19.4±3.5 | 42.4±6.5 | 12.6±0.2 | 126.1±2.6 | 99.7±0.6 | 107.7±3.1 | 19.9±0.7 | 91.8±0.9 |
| 7 (Atropine) | 224.0±16.0 | 17.3±3.8 | 48.5±7.5 | 13.0±0.2 | 130.2±2.4 | 100.0±0.0 | 99.3±6.8 | 19.0±0.6 | 88.8±3.3 |
| 8 (Scopolamine) | 216.3±13.2 | 23.5±2.5 | 34.0±1.7 | 11.9±1.1 | 119.9±11.4 | 99.7±0.6 | 97.3±6.7 | 18.2±1.0 | 91.0±1.3 |
| 9 (MeOH control) | 253.2±14.4 | 20.6±6.6 | 48.4±15.5 | 12.6±1.0 | 125.6±10.2 | 100.0±0.0 | 101.0±5.3 | 18.4±1.8 | 93.8±6.3 |

**Table S9:** Mass balance of BSF and LMW starting feed, diet residue and larvae, calculated on DM basis, in g. Mass ratios are calculated between feed, larvae and diet residue. N=3.

| **BSF Phase 1**  **Treatment** | **BSF starting feed phase 1**  **(g)** | **BSF diet residue phase 1**  **(g)** | **BSF larvae phase 1**  **(g)** | **Mass ratio**  **diet residue/ starting feed** | **Mass ratio larvae/**  **starting feed** | **Mass ratio diet residue + larvae/starting feed** | **Mass ratio larvae/diet residue** |
| --- | --- | --- | --- | --- | --- | --- | --- |
| 1 (*J. vulgaris*) | 316 | 159±9 | 62±4 | 0.515±0.030 | 0.195±0.011 | 0.710±0.021 | 0.381±0.042 |
| 2 (*S. vulgaris*) | 317 | 163±25 | 67±6 | 0.545±0.078 | 0.210±0.020 | 0.755±0.059 | 0.394±0.096 |
| 3 (*S. inaequidens*) | 317 | 212±22 | 62±10 | 0.663±0.071 | 0.197±0.031 | 0.860±0.040 | 0.303±0.078 |
| 4 (*E. vulgare*) | 317 | 167±14 | 62±1 | 0.529±0.043 | 0.196±0.005 | 0.725±0.039 | 0.373±0.040 |
| 5 (*H. europaeum*) | 317 | 168±12 | 67±2 | 0.542±0.037 | 0.211±0.007 | 0.752±0.030 | 0.391±0.042 |
| 6 (5 Herbs mix) | 318 | 162±11 | 68±2 | 0.507±0.035 | 0.215±0.007 | 0.722±0.042 | 0.424±0.015 |
| 7 (Atropine) | 319 | 151±7 | 66±5 | 0.478±0.020 | 0.208±0.016 | 0.687±0.004 | 0.437±0.052 |
| 8 (Scopolamine) | 320 | 169±12 | 65±4 | 0.516±0.039 | 0.203±0.012 | 0.719±0.049 | 0.395±0.017 |
| 9 (MeOH control) | 320 | 133±11 | 75±4 | 0.433±0.033 | 0.235±0.014 | 0.668±0.021 | 0.546±0.070 |

| **BSF Phase 2**  **Treatment** | **BSF starting feed phase 2**  **(g)** | **BSF diet residue phase 2**  **(g)** | **BSF larvae phase 2**  **(g)** | **Mass ratio**  **diet residue/ starting feed** | **Mass ratio larvae/**  **starting feed** | **Mass ratio diet residue + larvae/starting feed** | **Mass ratio larvae/diet residue** |
| --- | --- | --- | --- | --- | --- | --- | --- |
| 1 (*J. vulgaris*) | 16.1 | 2.4±0.2 | 4.0±0.1 | 0.150±0.013 | 0.248±0.009 | 0.397±0.014 | 1.665±0.161 |
| 2 (*S. vulgaris*) | 16.1 | 2.5±0.5 | 4.0±0.4 | 0.153±0.032 | 0.250±0.022 | 0.403±0.046 | 1.666±0.276 |
| 3 (*S. inaequidens*) | 16.1 | 3.0±0.2 | 3.8±0.0 | 0.187±0.013 | 0.233±0.002 | 0.420±0.014 | 1.251±0.094 |
| 4 (*E. vulgare*) | 16.1 | 2.4±0.2 | 4.2±0.1 | 0.146±0.014 | 0.260±0.007 | 0.406±0.020 | 1.783±0.138 |
| 5 (*H. europaeum*) | 16.1 | 2.4±0.0 | 3.9±0.0 | 0.147±0.002 | 0.241±0.001 | 0.388±0.001 | 1.634±0.029 |
| 6 (5 Herbs mix) | 16.1 | 2.7±0.1 | 3.9±0.1 | 0.169±0.004 | 0.241±0.007 | 0.410±0.005 | 1.431±0.069 |
| 7 (Atropine) | 16.1 | 2.6±0.2 | 4.1±0.1 | 0.162±0.012 | 0.253±0.007 | 0.415±0.013 | 1.569±0.130 |
| 8 (Scopolamine) | 16.1 | 2.7±0.4 | 4.0±0.3 | 0.167±0.025 | 0.246±0.020 | 0.412±0.006 | 1.512±0.357 |
| 9 (MeOH control) | 16.1 | 2.4±0.3 | 4.0±0.2 | 0.146±0.021 | 0.249±0.009 | 0.395±0.016 | 1.738±0.294 |

| **LMW**  **Treatment** | **LMW starting feed**  **(g)** | **LMW diet residue**  **(g)** | **LMW larvae**  **(g)** | **Mass ratio**  **diet residue/ starting feed** | **Mass ratio larvae/**  **starting feed** | **Mass ratio diet residue + larvae/starting feed** | **Mass ratio larvae/diet residue** |
| --- | --- | --- | --- | --- | --- | --- | --- |
| 1 (*J. vulgaris*) | 229 | 200±3 | 28.8±0.8 | 0.874±0.015 | 0.126±0.003 | 0.999±0.013 | 0.144±0.006 |
| 2 (*S. vulgaris*) | 229 | 196±1 | 30.0±0.4 | 0.855±0.004 | 0.131±0.002 | 0.986±0.004 | 0.153±0.002 |
| 3 (*S. inaequidens*) | 229 | 205±2 | 28.1±0.6 | 0.893±0.011 | 0.122±0.003 | 1.015±0.010 | 0.137±0.004 |
| 4 (*E. vulgare*) | 229 | 200±6 | 29.0±1.4 | 0.872±0.025 | 0.126±0.006 | 0.998±0.031 | 0.145±0.003 |
| 5 (*H. europaeum*) | 229 | 208±5 | 29.0±1.1 | 0.906±0.020 | 0.127±0.005 | 1.032±0.017 | 0.140±0.008 |
| 6 (5 Herbs mix) | 229 | 195±2 | 32.1±0.4 | 0.852±0.008 | 0.140±0.002 | 0.993±0.008 | 0.164±0.003 |
| 7 (Atropine) | 229 | 197±6 | 30.5±1.6 | 0.858±0.027 | 0.133±0.007 | 0.992±0.022 | 0.155±0.012 |
| 8 (Scopolamine) | 229 | 196±7 | 30.6±2.0 | 0.855±0.029 | 0.133±0.009 | 0.988±0.020 | 0.157±0.016 |
| 9 (MeOH control) | 229 | 193±3 | 31.4±1.3 | 0.843±0.013 | 0.137±0.006 | 0.980±0.009 | 0.163±0.009 |

**Table S10:** Plant toxin mass balance in BSF and LMW starting feed, diet residue and larvae, on DM basis, in µg total toxin. N=3.

| **BSF phase 1**  **Treatment** | **BSF starting feed phase 1**  **(µg)** | **BSF diet residue phase 1**  **(µg)** | **BSF larvae phase 1**  **(µg)** | **Ratio diet residue/ starting feed** | **Ratio larvae/starting feed** | **Ratio diet residue + larvae/starting feed** | **Ratio larvae/diet residue** |
| --- | --- | --- | --- | --- | --- | --- | --- |
| 1 (*J. vulgaris*) | 1441 | 249±14 | 8.8±0.5 | 0.154±0.009 | 0.005±0.000 | 0.160±0.009 | 0.035±0.004 |
| 2 (*S. vulgaris*) | 2268 | 1344±193 | 46.2±4.3 | 0.528±0.076 | 0.018±0.002 | 0.546±0.074 | 0.035±0.009 |
| 3 (*S. inaequidens*) | 14899 | 8049±834 | 341±55 | 0.481±0.050 | 0.020±0.003 | 0.502±0.047 | 0.043±0.011 |
| 4 (*E. vulgare*) | 1448 | 340±28 | 21.7±0.5 | 0.209±0.017 | 0.013±0.000 | 0.222±0.017 | 0.064±0.007 |
| 5 (*H. europaeum*) | 6014 | 2025±141 | 119±4.2 | 0.296±0.021 | 0.017±0.001 | 0.313±0.020 | 0.059±0.006 |
| 6 (5 Herbs mix) | 5407 | 1579±107 | 62.8±2.0 | 0.260±0.018 | 0.010±0.000 | 0.270±0.018 | 0.040±0.001 |
| 7 (Atropine) | 1037 | 344±16 | 14.3±1.0 | 0.305±0.014 | 0.013±0.001 | 0.317±0.013 | 0.042±0.005 |
| 8 (Scopolamine) | 336 | 267±21 | 7.3±0.4 | 0.713±0.053 | 0.020±0.001 | 0.733±0.054 | 0.028±0.002 |
| 9 (MeOH control) | 0.0 | 0.0 | 0.0 | - | - | - | - |

| **BSF phase 2**  **Treatment** | **BSF starting feed, phase 2**  **(µg)** | **BSF diet residue phase 2**  **(µg)** | **BSF larvae phase 2**  **(µg)** | **Ratio diet residue/ starting feed** | **Ratio larvae/starting feed** | **Ratio diet residue + larvae/starting feed** | **Ratio larvae/diet residue** |
| --- | --- | --- | --- | --- | --- | --- | --- |
| 1 (*J. vulgaris*) | 75.3 | 2.3±0.4 | 0.22±0.01 | 0.030±0.005 | 0.0029±0.0002 | 0.033±0.005 | 0.099±0.021 |
| 2 (*S. vulgaris*) | 119 | 5.4±0.7 | 0.21±0.02 | 0.046±0.006 | 0.0017±0.0002 | 0.047±0.006 | 0.038±0.002 |
| 3 (*S. inaequidens*) | 787 | 77.2±22.7 | 1.75±0.33 | 0.098±0.029 | 0.0022±0.0004 | 0.100±0.029 | 0.024±0.005 |
| 4 (*E. vulgare*) | 76.3 | 2.5±1.1 | 0.10±0.02 | 0.033±0.015 | 0.0014±0.0003 | 0.034±0.015 | 0.044±0.009 |
| 5 (*H. europaeum*) | 320 | 21.8±2.0 | 4.29±0.50 | 0.068±0.006 | 0.0134±0.0016 | 0.082±0.008 | 0.196±0.007 |
| 6 (5 Herbs mix) | 285 | 17.8±4.9 | 1.34±0.18 | 0.063±0.017 | 0.0047±0.0007 | 0.067±0.016 | 0.080±0.028 |
| 7 (Atropine) | 52.5 | 3.6±0.7 | 0.12±0.01 | 0.068±0.014 | 0.0024±0.0002 | 0.070±0.014 | 0.036±0.009 |
| 8 (Scopolamine) | 17.0 | 5.4±0.3 | 0.13±0.00 | 0.317±0.015 | 0.0075±0.0002 | 0.324±0.015 | 0.024±0.001 |
| 9 (MeOH control) | 0.0 | 0.0 | 0.0 | - | - | - | - |

| **LMW**  **Treatment** | **LMW starting feed (µg)** | **LMW diet residue (µg)** | **LMW larvae (µg)** | **Ratio diet residue/ starting feed** | **Ratio larvae/starting feed** | **Ratio diet residue + larvae/starting feed** | **Ratio larvae/diet residue** |
| --- | --- | --- | --- | --- | --- | --- | --- |
| 1 (*J. vulgaris*) | 1104 | 679±45 | 1.19±0.12 | 0.615±0.041 | 0.0011±0.0001 | 0.616±0.041 | 0.0018±0.0003 |
| 2 (*S. vulgaris*) | 1931 | 1337±115 | 0.99±0.18 | 0.692±0.059 | 0.0005±0.0001 | 0.693±0.060 | 0.0007±0.0001 |
| 3 (*S. inaequidens*) | 10537 | 8443±709 | 6.56±0.92 | 0.801±0.067 | 0.0006±0.0001 | 0.802±0.067 | 0.0008±0.0001 |
| 4 (*E. vulgare*) | 1029 | 789±14 | 0.29±0.09 | 0.767±0.013 | 0.0003±0.0001 | 0.767±0.013 | 0.0004±0.0001 |
| 5 (*H. europaeum*) | 4099 | 3749±211 | 26.01±2.35 | 0.915±0.052 | 0.0063±0.0006 | 0.921±0.051 | 0.0070±0.0010 |
| 6 (5 Herbs mix) | 4257 | 2715±314 | 8.20±0.52 | 0.638±0.074 | 0.0019±0.0001 | 0.640±0.074 | 0.0031±0.0005 |
| 7 (Atropine) | 709 | 378±13 | 0.43±0.05 | 0.533±0.018 | 0.0006±0.0001 | 0.533±0.018 | 0.0011±0.0001 |
| 8 (Scopolamine) | 198 | 185±0.2 | 0.20±0.04 | 0.934±0.001 | 0.0010±0.0002 | 0.935±0.001 | 0.0011±0.0002 |
| 9 (MeOH control) | 0 | 0.0 | 0.0 | - | - | - | - |

**Table S11:** Plant toxin bioaccumulation factors in LMW, BSF phase 1 and phase 2 larvae. Bioaccumulation factors are calculated by dividing the concentration in larvae by the concentration in the diet residue, calculated on a DM basis.

| **BSF larvae phase 1** | **1**  **(*J. vulgaris*)** | **2**  **(*S. vulgaris*)** | **3**  **(*S. inaequidens*)** | **4**  **(*E. vulgare*)** | **5**  **(*H. europaeum*)** | **6**  **(5 Herbs mix)** | **7**  **(Atropine)** | **8 (Scopolamine)** |
| --- | --- | --- | --- | --- | --- | --- | --- | --- |
| Erucifoline | 0.096±0.036 |  |  |  |  | 0.102±0.006 |  |  |
| Integerrimine | 0.089±0.011 | 0.082±0.006 | 0.155±0.004 |  |  | 0.095±0.004 |  |  |
| Jacobine | 0.123±0.047 |  |  |  |  | 0.142±0.005 |  |  |
| Jacoline | 0.101±0.041 |  |  |  |  |  |  |  |
| Retrorsine | 0.063±0.033 | 0.096±0.011 | 0.152±0.009 |  |  | 0.076±0.008 |  |  |
| Riddelliine |  | 0.073±0.001 |  |  |  |  |  |  |
| Sceleratine |  |  | 0.206±0.018 |  |  |  |  |  |
| Senecionine | 0.098±0.010 | 0.092±0.007 | 0.151±0.007 |  |  | 0.086±0.016 |  |  |
| Seneciphylline | 0.093±0.006 | 0.090±0.000 |  |  |  | 0.094±0.000 |  |  |
| Senecivernine |  | 0.078±0.036 | 0.123±0.001 |  |  | 0.071±0.003 |  |  |
| Senkirkine |  |  | 0.128±0.006 |  |  | 0.076±0.004 |  |  |
| Spartioidine |  | 0.092±0.005 |  |  |  |  |  |  |
| Usaramine |  | 0.075±0.012 | 0.147±0.008 |  |  | 0.086±0.007 |  |  |
| Echimidine |  |  |  | 0.164±0.003 |  | 0.078±0.004 |  |  |
| Echinatine |  |  |  |  | 0.160±0.004 | 0.188±0.004 |  |  |
| Europine |  |  |  |  | 0.164±0.002 | 0.196±0.002 |  |  |
| Heliosupine |  |  |  |  | 0.067±0.008 |  |  |  |
| Heliotrine |  |  |  |  | 0.148±0.010 | 0.116±0.000 |  |  |
| Lasiocarpine |  |  |  |  | 0.077±0.001 | 0.066±0.001 |  |  |
| Rinderine |  |  |  |  | 0.186±0.001 | 0.206±0.001 |  |  |
| Atropine |  |  |  |  |  |  | 0.094±0.007 |  |
| Scopolamine |  |  |  |  |  |  |  | 0.072±0.007 |
| Retrorsine N-oxide |  |  | 0.102±0.041 |  |  |  |  |  |
| Europine N-oxide |  |  |  |  | 5.47±4.26 |  |  |  |
| Heliotrine N-oxide |  |  |  |  | 6.41±5.00 |  |  |  |
| Echimidine N-oxide |  |  |  | 2.17±0.13 |  |  |  |  |
| Lasiocarpine N-oxide |  |  |  |  | 2.69±1.69 |  |  |  |
| PA FBs | 0.094±0.022 | 0.090±0.004 | 0.143±0.006 | 0.161±0.004 | 0.141±0.003 | 0.095±0.003 |  |  |
| PA NOs |  |  | 0.102±0.022 | 2.17±0.13 | 4.97±3.73 |  |  |  |
| Overall | 0.094±0.022 | 0.090±0.004 | 0.143±0.006 | 0.167±0.004 | 0.151±0.001 | 0.094±0.003 | 0.095±0.007 | 0.072±0.007 |

| **BSF larvae phase 2** | **1**  **(*J. vulgaris*)** | **2**  **(*S. vulgaris*)** | **3**  **(*S. inaequidens*)** | **4**  **(*E. vulgare*)** | **5**  **(*H. europaeum*)** | **6**  **(5 Herbs mix)** | **7**  **(Atropine)** | **8 (Scopolamine)** |
| --- | --- | --- | --- | --- | --- | --- | --- | --- |
| Erucifoline | 0.072±0.015 |  |  |  |  |  |  |  |
| Integerrimine |  | 0.013±0.004 | 0.009±0.002 |  |  | 0.012±0.004 |  |  |
| Jacobine | 0.124±0.048 |  |  |  |  |  |  |  |
| Jacoline | 0.183±0.058 |  |  |  |  |  |  |  |
| Retrorsine | 0.048±0.008 | 0.038±0.004 | 0.021±0.005 |  |  | 0.033±0.012 |  |  |
| Riddelliine |  | 0.041±0.017 |  |  |  |  |  |  |
| Sceleratine |  |  | 0.063±0.026 |  |  |  |  |  |
| Senecionine | 0.034±0.008 | 0.028±0.011 | 0.018±0.005 |  |  | 0.024±0.012 |  |  |
| Seneciphylline | 0.030±0.006 | 0.019±0.002 |  |  |  | 0.019±0.008 |  |  |
| Senecivernine |  |  | 0.016±0.003 |  |  | 0.021±0.008 |  |  |
| Senkirkine |  |  | 0.010±0.000 |  |  | 0.013±0.003 |  |  |
| Usaramine |  |  | 0.031±0.010 |  |  | 0.052±0.017 |  |  |
| Echimidine |  |  |  | 0.025±0.004 |  | 0.025±0.012 |  |  |
| Echinatine |  |  |  |  | 0.114±0.002 | 0.150±0.047 |  |  |
| Europine |  |  |  |  | 0.158±0.011 | 0.141±0.027 |  |  |
| Heliotrine |  |  |  |  | 0.085±0.008 | 0.082±0.030 |  |  |
| Lasiocarpine |  |  |  |  | 0.019±0.002 | 0.020±0.004 |  |  |
| Rinderine |  |  |  |  | 0.145±0.006 | 0.131±0.031 |  |  |
| Atropine |  |  |  |  |  |  | 0.023±0.007 |  |
| Scopolamine |  |  |  |  |  |  |  | 0.020±0.004 |
| Overall | 0.060±0.015 | 0.023±0.003 | 0.019±0.004 | 0.024±0.001 | 0.120±0.006 | 0.056±0.018 | 0.023±0.007 | 0.020±0.004 |

| **LMW larvae** | **1**  **(*J. vulgaris*)** | **2**  **(*S. vulgaris*)** | **3**  **(*S. inaequidens*)** | **4**  **(*E. vulgare*)** | **5**  **(*H. europaeum*)** | **6**  **(5 Herbs mix)** | **7**  **(Atropine)** | **8 (Scopolamine)** |
| --- | --- | --- | --- | --- | --- | --- | --- | --- |
| Erucifoline | 0.015±0.002 |  |  |  |  |  |  |  |
| Integerrimine |  |  | 0.009±0.001 |  |  | 0.007±0.002 |  |  |
| Jacobine | 0.026±0.001 |  |  |  |  |  |  |  |
| Jacoline | 0.072±0.004 |  |  |  |  | 0.124±0.019 |  |  |
| Jaconine | 0.027±0.003 |  |  |  |  |  |  |  |
| Retrorsine |  | 0.007±0.001 | 0.008±0.001 |  |  | 0.010±0.002 |  |  |
| Riddelliine |  | 0.007±0.006 |  |  |  |  |  |  |
| Sceleratine |  |  | 0.034±0.008 |  |  | 0.056±0.008 |  |  |
| Senecionine | 0.011±0.001 | 0.006±0.001 | 0.010±0.001 |  |  | 0.013±0.003 |  |  |
| Seneciphylline | 0.021±0.006 | 0.013±0.000 |  |  |  | 0.023±0.005 |  |  |
| Senecivernine |  |  | 0.006±0.001 |  |  | 0.007±0.001 |  |  |
| Senkirkine |  |  | 0.004±0.001 |  |  |  |  |  |
| Spartioidine |  |  |  |  |  |  |  |  |
| Usaramine |  |  | 0.011±0.001 |  |  | 0.015±0.003 |  |  |
| Echimidine |  |  |  | 0.004±0.002 |  | 0.009±0.003 |  |  |
| Echinatine |  |  |  |  | 0.206±0.044 |  |  |  |
| Europine |  |  |  |  | 0.454±0.059 | 0.531±0.102 |  |  |
| Heliosupine |  |  |  |  |  |  |  |  |
| Heliotrine |  |  |  |  | 0.042±0.010 | 0.049±0.014 |  |  |
| Lasiocarpine |  |  |  |  | 0.009±0.001 |  |  |  |
| Rinderine |  |  |  |  | 0.228±0.053 | 0.249±0.057 |  |  |
| Atropine |  |  |  |  |  |  | 0.007±0.001 |  |
| Scopolamine |  |  |  |  |  |  |  | 0.006±0.001 |
| Integerrimine N-oxide |  |  | 0.004±0.001 |  |  |  |  |  |
| Retrorsine N-oxide |  |  | 0.004±0.001 |  |  | 0.002±0.001 |  |  |
| Senecionine N-oxide |  | 0.005±0.004 | 0.009±0.002 |  |  |  |  |  |
| Senecivernine N-oxide |  |  | 0.004±0.001 |  |  |  |  |  |
| Usaramine N-oxide |  |  | 0.004±0.001 |  |  |  |  |  |
| Europine N-oxide |  |  |  |  | 0.007±0.001 | 0.005±0.001 |  |  |
| Heliotrine N-oxide |  |  |  |  | 0.004±0.001 |  |  |  |
| Lasiocarpine N-oxide |  |  |  |  | 0.006±0.001 |  |  |  |
| PA FBs | 0.020±0.002 | 0.008±0.001 | 0.007±0.001 | 0.004±0.002 | 0.143±0.017 | 0.036±0.007 |  |  |
| PA NOs |  | 0.001±0.001 | 0.004±0.001 |  | 0.005±0.001 | 0.001±0.000 |  |  |
| Overall | 0.012±0.001 | 0.005±0.001 | 0.006±0.001 | 0.003±0.001 | 0.050±0.006 | 0.019±0.006 | 0.007±0.001 | 0.006±0.001 |

**Table S12:** Plant toxin transfer factors in LMW, BSF phase 1 and phase 2 larvae. Transfer factors are calculated by dividing the amount of toxin present in the larvae divided by the amount of toxin present in the diet portion that has been consumed by the larvae. The toxin concentration in the diet residue is taken as reference point for calculation of the transfer factors and it is assumed that the concentration in the remaining diet residue was not affected by the larvae.

| **BSF larvae phase 1** | **1**  **(*J. vulgaris*)** | **2**  **(*S. vulgaris*)** | **3**  **(*S. inaequidens*)** | **4**  **(*E. vulgare*)** | **5**  **(*H. europaeum*)** | **6**  **(5 Herbs mix)** | **7**  **(Atropine)** | **8 (Scopolamine)** |
| --- | --- | --- | --- | --- | --- | --- | --- | --- |
| Erucifoline | 0.038±0.001 |  |  |  |  | 0.045±0.005 |  |  |
| Integerrimine | 0.036±0.001 | 0.038±0.003 | 0.091±0.006 |  |  | 0.042±0.004 |  |  |
| Jacobine | 0.048±0.002 |  |  |  |  | 0.062±0.007 |  |  |
| Jacoline | 0.040±0.001 |  |  |  |  |  |  |  |
| Retrorsine | 0.025±0.001 | 0.044±0.004 | 0.089±0.006 |  |  | 0.033±0.001 |  |  |
| Riddelliine |  | 0.034±0.003 |  |  |  |  |  |  |
| Sceleratine |  |  | 0.121±0.008 |  |  |  |  |  |
| Senecionine | 0.039±0.001 | 0.043±0.003 | 0.089±0.006 |  |  | 0.031±0.003 |  |  |
| Seneciphylline | 0.037±0.001 | 0.042±0.003 |  |  |  | 0.041±0.004 |  |  |
| Senecivernine |  | 0.036±0.003 | 0.072±0.005 |  |  | 0.031±0.003 |  |  |
| Senkirkine |  |  | 0.075±0.005 |  |  | 0.033±0.004 |  |  |
| Spartioidine |  | 0.043±0.003 |  |  |  |  |  |  |
| Usaramine |  | 0.035±0.003 | 0.087±0.006 |  |  | 0.033±0.004 |  |  |
| Echimidine |  |  |  | 0.069±0.005 |  | 0.038±0.002 |  |  |
| Echinatine |  |  |  |  | 0.070±0.003 | 0.091±0.002 |  |  |
| Europine |  |  |  |  | 0.074±0.003 | 0.082±0.009 |  |  |
| Heliosupine |  |  |  |  | 0.031±0.001 |  |  |  |
| Heliotrine |  |  |  |  | 0.068±0.003 | 0.051±0.005 |  |  |
| Lasiocarpine |  |  |  |  | 0.035±0.002 | 0.029±0.003 |  |  |
| Rinderine |  |  |  |  | 0.086±0.004 | 0.090±0.010 |  |  |
| Atropine |  |  |  |  |  |  | 0.036±0.001 |  |
| Scopolamine |  |  |  |  |  |  |  | 0.030±0.004 |
| Overall | 0.038±0.001 | 0.042±0.003 | 0.040±0.005 | 0.069±0.005 | 0.065±0.003 | 0.041±0.004 | 0.038±0.001 | 0.030±0.004 |

| **BSF larvae phase 2** | **1**  **(*J. vulgaris*)** | **2**  **(*S. vulgaris*)** | **3**  **(*S. inaequidens*)** | **4**  **(*E. vulgare*)** | **5**  **(*H. europaeum*)** | **6**  **(5 Herbs mix)** | **7**  **(Atropine)** | **8 (Scopolamine)** |
| --- | --- | --- | --- | --- | --- | --- | --- | --- |
| Erucifoline | 0.021±0.004 |  |  |  |  |  |  |  |
| Integerrimine |  | 0.004±0.001 | 0.003±0.001 |  |  | 0.003±0.001 |  |  |
| Jacobine | 0.036±0.013 |  |  |  |  |  |  |  |
| Jacoline | 0.053±0.016 |  |  |  |  |  |  |  |
| Retrorsine | 0.014±0.002 | 0.011±0.001 | 0.006±0.001 |  |  | 0.010±0.004 |  |  |
| Riddelliine |  | 0.012±0.004 |  |  |  |  |  |  |
| Sceleratine |  |  | 0.018±0.007 |  |  |  |  |  |
| Senecionine | 0.010±0.002 | 0.008±0.004 | 0.005±0.001 |  |  | 0.007±0.003 |  |  |
| Seneciphylline | 0.009±0.001 | 0.006±0.001 |  |  |  | 0.006±0.003 |  |  |
| Senecivernine |  |  | 0.005±0.001 |  |  | 0.006±0.002 |  |  |
| Senkirkine |  |  | 0.003±0.000 |  |  | 0.004±0.001 |  |  |
| Usaramine |  |  | 0.009±0.003 |  |  | 0.015±0.005 |  |  |
| Echimidine |  |  |  | 0.007±0.001 |  | 0.007±0.004 |  |  |
| Echinatine |  |  |  |  | 0.032±0.000 | 0.044±0.014 |  |  |
| Europine |  |  |  |  | 0.045±0.003 | 0.041±0.009 |  |  |
| Heliosupine |  |  |  |  |  |  |  |  |
| Heliotrine |  |  |  |  | 0.024±0.002 | 0.024±0.009 |  |  |
| Lasiocarpine |  |  |  |  | 0.006±0.000 | 0.006±0.001 |  |  |
| Rinderine |  |  |  |  | 0.041±0.002 | 0.038±0.010 |  |  |
| Atropine |  |  |  |  |  |  | 0.007±0.002 |  |
| Scopolamine |  |  |  |  |  |  |  | 0.005±0.001 |
| Overall | 0.017±0.004 | 0.007±0.001 | 0.005±0.001 | 0.007±0.001 | 0.034±0.002 | 0.016±0.001 | 0.007±0.002 | 0.005±0.001 |

| **LMW larvae** | **1**  **(*J. vulgaris*)** | **2**  **(*S. vulgaris*)** | **3**  **(*S. inaequidens*)** | **4**  **(*E. vulgare*)** | **5**  **(*H. europaeum*)** | **6**  **(5 Herbs mix)** | **7**  **(Atropine)** | **8 (Scopolamine)** |
| --- | --- | --- | --- | --- | --- | --- | --- | --- |
| Erucifoline | 0.015±0.001 |  |  |  |  |  |  |  |
| Integerrimine |  |  | 0.010±0.001 |  |  | 0.006±0.002 |  |  |
| Jacobine | 0.026±0.003 |  |  |  |  |  |  |  |
| Jacoline | 0.072±0.006 |  |  |  |  | 0.118±0.022 |  |  |
| Retrorsine |  | 0.006±0.001 | 0.010±0.001 |  |  | 0.009±0.002 |  |  |
| Riddelliine |  | 0.006±0.005 |  |  |  |  |  |  |
| Sceleratine |  |  | 0.039±0.010 |  |  | 0.053±0.010 |  |  |
| Senecionine | 0.010±0.000 | 0.005±0.001 | 0.011±0.002 |  |  | 0.012±0.004 |  |  |
| Seneciphylline | 0.020±0.006 | 0.011±0.000 |  |  |  | 0.022±0.006 |  |  |
| Senecivernine |  |  | 0.007±0.001 |  |  | 0.007±0.002 |  |  |
| Senkirkine |  |  | 0.004±0.001 |  |  |  |  |  |
| Usaramine |  |  | 0.012±0.002 |  |  | 0.014±0.004 |  |  |
| Echimidine |  |  |  | 0.005±0.002 |  | 0.008±0.003 |  |  |
| Echinatine |  |  |  |  | 0.285±0.085 |  |  |  |
| Europine |  |  |  |  | 0.630±0.170 | 0.508±0.116 |  |  |
| Heliosupine |  |  |  |  |  |  |  |  |
| Heliotrine |  |  |  |  | 0.058±0.018 | 0.047±0.015 |  |  |
| Lasiocarpine |  |  |  |  | 0.012±0.001 |  |  |  |
| Rinderine |  |  |  |  | 0.322±0.119 | 0.239±0.064 |  |  |
| Atropine |  |  |  |  |  |  | 0.007±0.001 |  |
| Scopolamine |  |  |  |  |  |  |  | 0.006±0.002 |
| Integerrimine N-oxide |  |  | 0.005±0.001 |  |  |  |  |  |
| Retrorsine N-oxide |  |  | 0.005±0.001 |  |  | 0.002±0.001 |  |  |
| Senecionine N-oxide |  | 0.004±0.004 | 0.010±0.002 |  |  |  |  |  |
| Senecivernine N-oxide |  |  | 0.004±0.001 |  |  |  |  |  |
| Usaramine N-oxide |  |  | 0.004±0.001 |  |  |  |  |  |
| Europine N-oxide |  |  |  |  | 0.010±0.002 | 0.005±0.001 |  |  |
| Heliotrine N-oxide |  |  |  |  | 0.006±0.001 |  |  |  |
| Lasiocarpine N-oxide |  |  |  |  | 0.008±0.000 |  |  |  |
|  |  |  |  |  |  |  |  |  |
| PA FBs | 0.020±0.001 | 0.008±0.001 | 0.008±0.001 | 0.005±0.002 | 0.198±0.050 | 0.035±0.008 |  |  |
| PA NOs |  | 0.001±0.001 | 0.005±0.001 |  | 0.007±0.001 | 0.001±0.000 |  |  |
| Overall | 0.012±0.001 | 0.004±0.001 | 0.007±0.001 | 0.003±0.001 | 0.069±0.018 | 0.018±0.004 | 0.007±0.001 | 0.006±0.002 |

Figure S1: Representative pyrrolizidine and tropane alkaloids present in Asteraceae, Boraginaceae or Solanaceae species.
